# CD44 variant exons induce chemoresistance by modulating cell death pathways

**DOI:** 10.3389/fcell.2025.1508577

**Published:** 2025-03-06

**Authors:** Maria Yanova, Evgeniya Stepanova, Diana Maltseva, Alexander Tonevitsky

**Affiliations:** ^1^ Faculty of Biology and Biotechnology, National Research University Higher School of Economics, Moscow, Russia; ^2^ Shemyakin-Ovchinnikov Institute of Bioorganic Chemistry, Russian Academy of Sciences, Moscow, Russia

**Keywords:** CD44 variant exon 6, CD44 variant exon 9, cancer, chemoresistance, apoptosis, ferroptosis, autophagy

## Abstract

Cancer chemoresistance presents a challenge in oncology, often leading to treatment failure and disease progression. CD44, a multifunctional cell surface glycoprotein, has garnered attention for its involvement in various aspects of cancer biology. Through alternative splicing, CD44 can form isoforms with the inclusion of only standard exons, typical for normal tissue, or with the addition of variant exons, frequently expressed in cancer tissue and associated with chemoresistance. The functions of CD44 involved in regulation of cancer signaling pathways are being actively studied, and the significance of specific variant exons in modulating cell death pathways, central to the response of cancer cells to chemotherapy, begins to become apparent. This review provides a comprehensive analysis of the association of CD44 variant exons/total CD44 with clinical outcomes of patients undergoing chemotherapy. The role of CD44 variant exons v6, v9 and others with a significant effect on patient chemotherapy outcomes by means of key cellular death pathways such as apoptosis, ferroptosis and autophagy modulation is further identified, and their impact on drug resistance is highlighted. An overview of clinical trials aimed at targeting variant exon-containing isoforms is provided, and possible directions for further development of CD44-targeted therapeutic strategies are discussed.

## 1 Introduction

Cancer ranks among the leading causes of death worldwide, claiming approximately 10 million lives in 2022, according to GLOBOCAN data (Bray et al., 2024). This disease is characterized by the uncontrolled proliferation of abnormal cells, their evasion of cellular checkpoints that generally regulate cell growth and death, and their invasion into surrounding tissues (Hanahan, 2022). The complexity and adaptability of cancer cells make the disease particularly challenging to treat. To date, chemotherapy is one of the most prevalent and effective methods in addition to surgery for the treatment of patients (Anand et al., 2023). Despite significant advancements in the development of chemotherapeutic agents, resistance to these treatments remains a major challenge. Chemoresistance has been observed for nearly all chemotherapeutic agents, including commonly used drugs such as doxorubicin, paclitaxel, 5-fluorouracil (5-FU), cisplatin, and gemcitabine (Mollaei et al., 2021). This chemoresistance is multifaceted and can be attributed to several factors, with deficiencies in programmed cell death (PCD) being one of them (Sazonova et al., 2024).

CD44, a cell surface transmembrane glycoprotein, is known to regulate tumor progression and cancer-associated molecular signaling pathways. Notably, CD44 variant exon-containing isoforms, absent in norma and appearing during tumorigenesis due to alternative splicing, can interact with various cell membrane receptors/intracellular proteins, influencing signaling that promotes cell survival. A recent review indicates that CD44 (including CD44 variant exon containing isoforms) is involved in MAPK, Hippo and PI3K/Akt signaling pathways to name a few (Xu et al., 2024). Cell survival signaling is tightly interconnected with the sensitivity of cells to chemotherapeutic drugs. Consequently, through the regulation of cell death signaling pathways, CD44 variant exon containing isoforms may be presented as targets for selective modulation of chemosensitivity. However, how particular CD44 exons are involved in cell death pathways modulation is still under question. Hence, this review explores the association of CD44 variant exon expression with clinical outcomes of patients undergoing chemotherapy, highlights their role in key cell death pathways regulation, provides critical analysis of therapeutic substances and clinical studies targeting CD44 and discusses directions for further development of CD44 targeted therapeutic strategies.

Importantly, among the nine variant exons of CD44 (v2-v10), our review focuses on three exons—v3, v6, and v9—that are associated with patient survival following chemotherapy. The molecular mechanisms underlying these associations have been described in relation to their biological function both *in vitro* and *in vivo* (with the exception of v3). Although the expression levels of variant exons v7 and v10 have been shown to correlate with patient survival, the existing literature on the molecular mechanisms of chemoresistance for these exons is limited. As a result, we do not cover them in this review.

## 2 Molecular structure and functional Basis of CD44 and its splicing variants

CD44, a non-kinase cell surface transmembrane glycoprotein, was described in 1989 by Stamenkovic et al., and two main forms of the protein were identified–a lymphoid form present in hematopoietic cells and an epithelial form weakly expressed in normal epithelium and abundantly expressed in carcinomas (Stamenkovic et al., 1989). CD44 activity is modulated by interaction with its ligands, mainly with hyaluronan (HA) (Aruffo et al., 1990).

**FIGURE 1:**
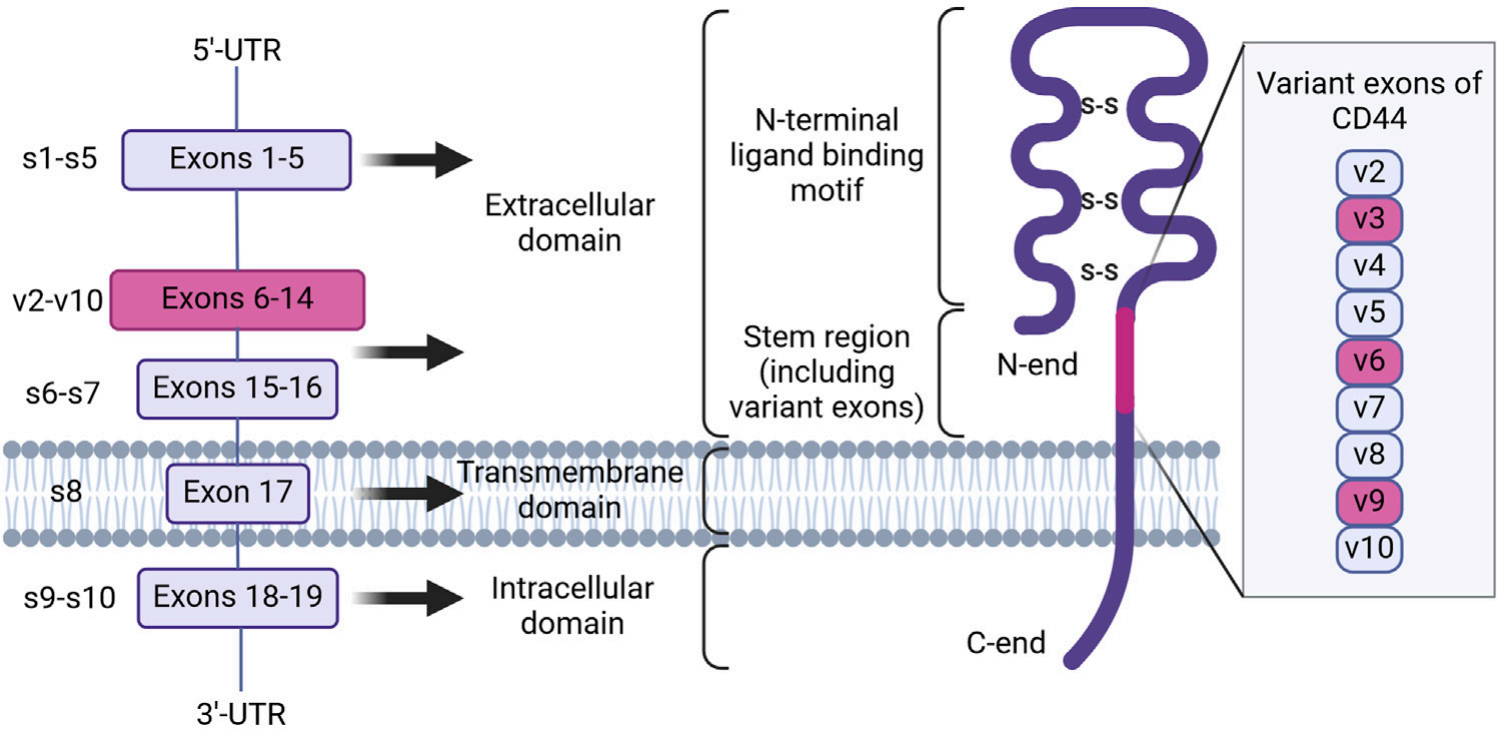
The genomic organization and protein structure of human CD44. CD44 human gene contains 19 exons. Alternative splicing results in the formation of CD44 standard and variant isoforms. CD44 protein consists of an intracellular and transmembrane domain, consisting of exons 18–19 and 17, and an extracellular domain, consisting of exons 1–16. Inclusion of variant exons (pink) to CD44 isoforms is observed during cancer progression and chemoresistance, with the latter being associated with variant exons v3, v6 and v9 (Created with Biorender.com).

Research has identified that CD44 is encoded by a highly conserved gene on the short arm of human chromosome 11 (Screaton et al., 1992). Nineteen exons are involved in the genomic organization of this molecule. As a result of alternative splicing and posttranslational modifications, at least 21 predicted CD44 isoforms are generated with 8 experimentally confirmed isoforms with different molecular weight (in the range of 85–250 kDa) and function (Gaiteiro et al., 2022). The smallest isoform, alternatively named hematopoietic or standard isoform 4 (CD44s, isoform 4), is ubiquitously expressed in vertebrate cells of mesenchymal origin, including leukocytes, fibroblasts and neuronal cells (Chen et al., 2020; Naor et al., 2008) and is translated from the first five (exons 1-5 or s1-5) and the last four (exons 15–17 and 19 or s15–17 and s19) exons (Figure 1). On the contrary, nine variant exons (exons 6–14 or v2-v10), located in the middle of the CD44 gene can be alternatively spliced and assembled with exons contained in CD44s, resulting in the formation of a plethora of CD44 variant isoforms, typical of cells of epithelial origin and several carcinomas. The mechanisms by which variant isoforms of CD44 are formed by alternative splicing are described in detail in the recent review (Maltseva and Tonevitsky, 2023).

At present, all CD44 isoforms belong to the type 1 transmembrane protein class and have a typical structure consisting of the extracellular domain (ECD), the transmembrane domain (TMD) and the cytoplasmic or intracellular domain (ICD) (Figure 1; Chen et al., 2020). The ECD, in turn, includes several regions necessary for sensoring, structural integrity and activation of CD44 protein. Firstly, the N-terminal ligand-binding motif or HA-binding domain (HABD), oriented into the extracellular matrix, is present in all isoforms of CD44 (both CD44s and CD44 variant isoforms), consists of constant exons 1-5 and forms a compactly-folded domain. HABD allows interaction with extracellular matrix components such as HA, osteopontin, fibronectin, collagen, growth factors, cytokines and matrix metalloproteinases in a glycosylation- and disulfide-bond reduction-dependent manner (Senbanjo and Chellaiah, 2017). Secondly, the variant region present in variant isoforms can vary significantly in length depending on what exons (6–14) are included in the mRNA after splicing (resulting, for example, in the formation of isoforms CD44v2-v10, CD44v3-v10, CD44v8-v10 or an isoform containing only one CD44v10 variant exon) and forms additional sites of interaction with growth factors and receptors on the plasma membrane, consequently expanding the functionality of CD44 (Liao et al., 2022). Lastly, the stem region is made up of exons 15 and 16. The TMD consists of exon 17 and contains a cysteine residue responsible for palmitoylation and consequent incorporation into lipid rafts of the cellular membrane. It also has been shown to provide a platform for CD44 oligomerization and coupling to adaptor proteins, receptor tyrosine kinases (RTKs) or nonreceptor protein-tyrosine kinases for consequent CD44 activation and signaling (Sun et al., 2020). The ICD contains a nuclear localization signal (NLS) for outside-in-cell signaling, consisting of two clusters of basic amino acids (^292^RRRCGQKKK^300^) (Skandalis, 2023). CD44 ICD can be cleaved from the membrane by presenilin-γ-secretase and transported into the nucleus, where it can act as a transcription factor through binding to consensus sequences found in the promoter regions of genes, including CD44 itself.

## 3 The expression profile of CD44 splice variants in cancer

**FIGURE 2:**
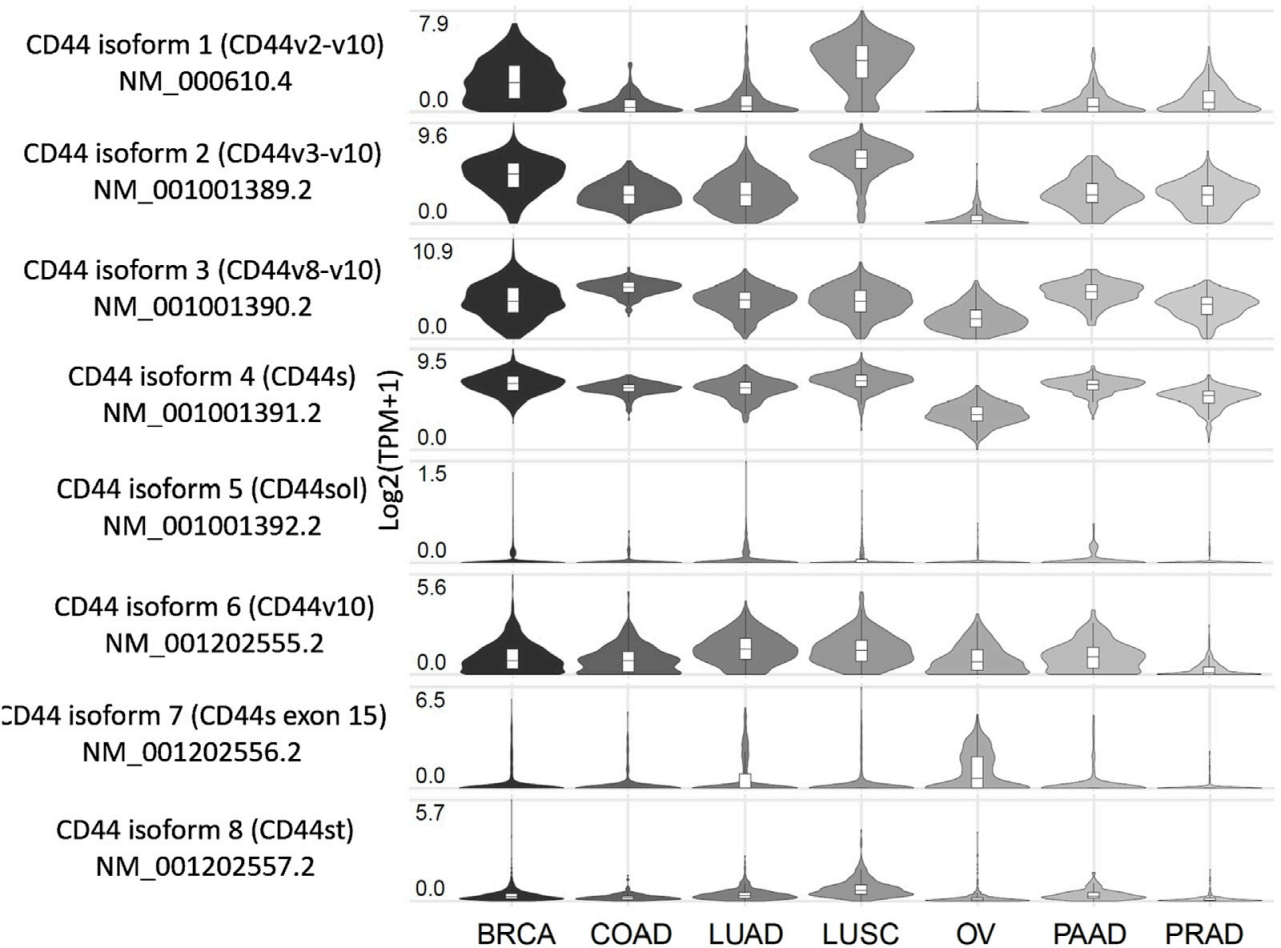
Expression profile of CD44 splice variants in patients with several tumor types, including the most common and those associated with the highest mortality. Created by www.gepia2.com.

Insight on the expression profile of different splice variants of CD44 can be of use for better understanding of cancer-specific molecular fingerprints and choosing an optimal cancer targeting strategy. In this section we compile what is known on the CD44 isoforms/exons expression profile from tumor samples and cell lines of clinically relevant cancers, as well as conduct an independent analysis using The Cancer Genome Atlas (TCGA) RNA sequencing (RNA-seq) data on CD44 splice isoforms derived from the GEPIA2 database (Figure 2; Tang et al., 2019). It should be noted that apart from experimentally determined CD44 isoforms in NCBI and UniProt databases used in our analysis, additional predicted CD44 isoforms exist (Supplementary Figure S1; Maltseva and Tonevitsky, 2023; Tang et al., 2019; Shi et al., 2024).

Below, we examine the results for several of the most common tumor types and those associated with the highest mortality rates (results for other tumor types can be found in Supplementary Figure S1). According to the GEPIA2 database (Figure 2), breast cancer (BRCA) patients predominantly express CD44 isoform 4 (CD44s), along with isoforms 1-3, which include exons v2-v10, and to a lesser extent, isoform 6 (CD44v10). Squamous cell lung cancer (LUSC) patients exhibit a similar expression profile to that of BRCA. Patients with colorectal (COAD), lung (LUAD), pancreatic (PAAD) and prostate (PRAD) cancers primarily express CD44s as well as isoforms 2 and 3, which include exons v3-v10. Notably, LUAD and PAAD patients also express isoform 6, which includes exon v10. Ovarian cancer patients (OV) tend to express CD44s and isoform 3 (CD44v8-v10), although it is worth noting that the expression levels are lower compared to those observed previously mentioned cancers.

According to literature, BRCA patients in accordance with TCGA data express isoforms 1–4 (Bànkfalvi et al., 1998; Tokue et al., 1998; Olsson et al., 2011; Table 1). Interestingly, in grade 1 invasive ductal carcinoma patients, more than 50% of tumor samples stain positive for CD44 variant exons v3, v5-v7 and v9 (CD44v3, CD44v5, CD44v6, CD44v7 and CD44v9), highlighting their potential as therapeutic targets for early grade ductal carcinoma (Bànkfalvi et al., 1998). However, as ductal carcinoma progresses to grades 2–3, an increase in tumor samples staining positive for CD44s and CD44v9 is observed, along with a decrease in samples staining positive for CD44v3, CD44v6 and CD44v7. This shift suggests that targeting CD44 variant exons becomes less feasible as the tumor advances (Bànkfalvi et al., 1998). Additionally, another study points to more than 50% of tumor samples stain positive for CD44v6, supporting its relevance (Tokue et al., 1998).

**TABLE 1:** The incidence and frequency profile of CD44 variant exons expression in several tumor types, including the most common and those associated with the highest mortality.

Incidence and frequency profile of CD44 variant exons in cancer										
CD44s	CD44v2	CD44v3	CD44v4	CD44v5	CD44v6	CD44v7	CD44v8	CD44v9	CD44v10	Source
Breast cancer: grade 1 (grades 2–3)										
40% (60%)	ND	50% (40%)	0% (15%)	80% (80%)	60% (30%)	50% (20%)	ND	70% (80%)	ND	Bànkfalvi et al. (1998)
ND	23%	ND			76%	ND				Tokue et al. (1998)
Lung cancer: LUSC (LUAD)										
58% (11%)	ND	78% (27%)	ND		91% (47%)	ND			44% (9%)	Tran et al. (1997)
95% (75%)	ND	90% (75%)	ND		75% (25%)	ND				Ariza et al. (1995)
Pancreatic cancer										
ND	38%	ND			50%	ND				Gotoda et al. (1998)
66%	ND	ND			50%	ND				Castella et al. (1996)
Prostate cancer										
68%	ND	13%	13%		36%	19%		68%	3%	Takahashi et al. (1998)
63%	ND	25%	ND		79%	ND				Aaltomaa et al. (2001)
Colorectal cancer										
ND					40%	ND	80%			Boman et al. (2023)
Ovarian cancer										
ND		22%	15%	ND	4%	ND		63%	ND	Cannistra et al. (1995)

Several studies on non-small cell lung cancer (NSCLC) have identified CD44v3, CD44v6 and CD44v10 in more than half of the patients with LUSC, whereas only CD44v6 and to a lesser extent CD44v3 are expressed in patients with LUAD (Tran et al., 1997; Table 1). These findings suggest that LUSC could potentially be targeted via several CD44 variant exons, while CD44 targeting in LUAD CD44 is predominantly restricted to CD44v3 (Tran et al., 1997). Additionally, in PAAD and PRAD patients, CD44v6 is expressed prominently. In PAAD, CD44v2 and CD44v3 are expressed to a lesser extent, whereas in PRAD, CD44v6 and CD44v9 are expressed. Notably, more than half of PRAD patients show positivity for CD44v6 and CD44v9 (Gotoda et al., 1998; Castella et al., 1996; Takahashi et al., 1998; Aaltomaa et al., 2001).

Research concerning CD44 splice variants expression profile has been conducted on TCGA patients and on colorectal cancer cell lines using TCGA RNA-seq data (Novosad et al., 2022; Everest‐Dass et al., 2024). It was identified that CD44 isoforms 2, 3 and 4 are predominantly expressed in colorectal cancer patients at the mRNA expression levels (Novosad et al., 2022). Moreover, CD44v6 and CD44v8-v10 were shown to be positively expressed in approximately more than half of the colorectal cancer tumors (Boman et al., 2023; Table 1).

In ovarian cancer, only CD44v9 was shown to be identified in more than half of the patient tumors (Cannistra et al., 1995). This is in accordance with TCGA data, since OVCA patients express only CD44s and CD44 isoform 3.

Overall, the cancers analyzed exhibit distinct expression profiles of CD44 isoforms/variant exons, based on our findings and immunohistochemical data available in the literature. According to TCGA data (GEPIA2), three distinct groups can be characterized by their CD44 isoform expression pattern: 1) cancers expressing CD44 isoforms 1-4 and consequently variant exons v2-v10, 2) cancers expressing CD44 isoforms 2-4 and variant exons v3-v10, and 3) cancers expressing only CD44 isoforms 3–4 (variant exons v8-v10). Moreover, the results of immunohistochemical analyses of expression profiles of CD44 isoforms/variant exons in tumor samples closely align with TCGA data. Differences in the CD44 isoform/variant exon expression profiles underscore the need for tailored CD44-targeting approaches.

## 4 Chemotherapy and cell death pathways modulation

Cell death, a crucial aspect of the response to chemotherapy, can occur through various mechanisms, each influencing chemoresistance: during tumor progression, the balance between pro-cell death and anti-cell death regulators is shifted towards survival by various escape mechanisms (D’Amico and De Amicis, 2024). Recent advances in research have significantly expanded our understanding of cell death processes, introducing several new mechanisms. The Nomenclature Committee on Cell Death (NCCD) has updated its guidelines to include these novel forms of cell death, reflecting the increasing complexity of the field (Galluzzi et al., 2018). Apart from well-known apoptosis and autophagy, types of cell death, such as ferroptosis, an iron-dependent form of cell death characterized by the accumulation of lipid peroxides, were included (Dixon and Olzmann, 2024).

Apoptosis, discovered in 1972, is a programmed and controlled process of cell self-destruction requiring specific cell signaling transduction (Wang et al., 2023). The use of anticancer drugs to induce cell death by apoptosis has been considered one of the most critical ways to kill cancer cells, partly due to the fact that it has been considered the only form of cell death. Many chemotherapy drugs are known to induce cellular death through apoptosis, including cisplatin, 5-FU and gemcitabine (Kaufmann and Earnshaw, 2000).

Ferroptosis is a nonapoptotic iron-dependent form of cell death driven by lipid peroxidation (Dixon et al., 2012). Ferroptosis has recently been proven to correlate with cancer therapy resistance. A plethora of studies have identified that regulation of ferroptosis could influence the efficacy of cancer treatment and reverse cancer therapy resistance (Friedmann Angeli et al., 2019; Wu et al., 2020). Some chemotherapy drugs have been found to induce ferroptosis, including cisplatin and gemcitabine (Zhou et al., 2024).

Autophagy is a biological process that allows cells to degrade and recycle proteins and organelles to maintain homeostasis and adapt to various stresses (Liu et al., 2023). Chemotherapeutic agents such as paclitaxel, docetaxel, cisplatin, doxorubicin and 5-FU are known to induce autophagy (Debnath et al., 2023). Moreover, research has shown that the stimulation of autophagy by paclitaxel and cisplatin prevents apoptosis of tumor cells and leads to cancer chemoresistance (Debnath et al., 2023; Li et al., 2021).

Overall, chemotherapy drugs commonly utilized in clinic have been shown to induce not only apoptosis, but additionally ferroptosis and autophagy. This may be useful in the sense that resistance to apoptosis is a widespread noted effect, and cells that are resistant to apoptosis remain sensitive to ferroptosis (dos Santos et al., 2023). Therefore, the combinational or seldom triggering of different cell death pathways may be helpful for chemoresistance alleviation.

According to a recent scoping review of clinical studies, the impact of CD44 expression on the efficacy of chemotherapy treatment primarily correlates with drugs such as 5-FU, cisplatin and docetaxel (Wu et al., 2024). These drugs are widely used to treat locally advanced or metastatic breast, colorectal, head and neck cancer, among others. However, how particular CD44 exons are involved in cell death pathways modulation is still under question. Hence, in our review, we focus on the effect exerted by variant exons that are alternative to independent isoforms since several isoforms structurally share the same exons and, therefore, could participate in similar exerted processes.

## 5 Total CD44 and chemotherapy resistance

### 5.1 Effect of total CD44 expression on chemotherapy treatment outcome/tumor response to chemotherapy in patients/animal studies

Firstly, the current section of our review will focus on total CD44 involvement in chemoresistance, since total CD44-mediated signaling may involve both standard and/or variant exon containing isoforms. Onwards, specific variant exons of CD44 will be discussed.

Overall, a significant negative effect of increased total CD44 expression on chemotherapy treatment outcome was observed across several types of cancers, including breast, colorectal and head and neck cancers measured typically through overall survival (OS) and recurrence-free survival (RFS) values, as well as through response to chemotherapy (Wu et al., 2024). Notably, several studies have identified no correlation or even a positive correlation with response to chemotherapy treatment outcome, which underlines the ambiguity of CD44’s relationship with chemotherapy outcomes (Wu et al., 2024). Such differences may be due to the variation of CD44 variant isoforms amongst different cancer types and the difference in activated survival pathways. Varying CD44 isoform/variant exon detection methods may also contribute to these differences.

In addition, the association between total CD44 overexpression/knockdown and chemoresistance *in vivo* has been studied (Table 2). Research indicates that CD44 knockdown (CD44^kd^) in prostate cancer xenografts significantly reduces tumor growth rate after continuous docetaxel treatment compared with control cells, suggesting that the combination of chemotherapy with CD44^kd^ can lead to a beneficial chemotherapy outcome (Hao et al., 2012).

**TABLE 2:** The association of CD44 variant exon expression levels with tumor response to chemotherapy in animal studies.

CD44 variant/only constant exons	Expression in cancer	Manipulation	Administered chemotherapy	Response *in vivo*	Source
Total CD44	Prostate cancer xenografts of cell line PC-3M-luc	Knockdown of total CD44	Docetaxel	- Significantly reduced tumor growth rate and smaller tumor volumes after continuous docetaxel treatment - Increased sensitivity to docetaxel	Hao et al. (2012)
CD44v6	Prostate cancer xenografts of cell line PC-3M-luc	Knockdown of CD44v6	Docetaxel	- Decreased tumor growth and volume - Decreased levels of phosphorylated mTOR and Akt proteins	Ni et al. (2020)
	Colon cancer xenografts of cell line SW948	Knockdown of CD44v6	FOLFOX-resistant cells	- Decreased tumor growth	Ghatak et al. (2021)
CD44v9	Gastric cancer xenografts of cell line MKN28	Overexpression of CD44v9	5-FU; SAS	- Decreased tumor volume	Miyoshi et al. (2018)
	Pancreatic cancer xenografts of cell line CFPAC-1	Isolation of CD44^high^ cells; knockdown of CD44 total in CD44^high^ cells	Gemcitabine	- More rapid tumor growth in CD44^high^ cells with/without gemcitabine treatment - Detection of CD44v9 in CD44^high^ cells - Increased gemcitabine sensitivity with CD44^kd^ in CD44^high^ cells	Zhao et al. (2016)

Literature analysis demonstrated a predominantly negative relationship between total CD44 expression levels and the clinical outcome of patients. Moreover, CD44^kd^ cancer xenografts *in vivo* significantly reduce tumor growth rate after continuous chemotherapy administration. Thus, total CD44, despite the presence of both standard and variant isoforms, seems to be involved in chemotherapy resistance.

### 5.2 Total CD44 biological functions in relation to cell death pathways

The underlying mechanisms of total CD44 in chemotherapy resistance are quite diverse, possibly due to its combination of several variant exon-containing isoforms and the standard isoform (Table 3). It has been shown to influence key cell death signaling pathways, including apoptosis, ferroptosis and autophagy.

**TABLE 3:** CD44 variant exon biological functions in relation to cell death pathways.

CD44 variant/only constant exons	Cell death pathway	Target	Cancer type	Manipulation	Effect *in vitro*	Source
Total CD44	Apoptosis	ERBB2	Colon cancer cell line HCT116	-	- Coimmunoprecipitation assay unveiled CD44/ERBB2 complex formation, with consequent dissociation upon HA oligomers addition - Regulatory subunits of PI3K p85 and p110a and ezrin are associated with CD44 - Hsp90/Cdc37, required for ERBB2 activity and stabilization interact with ERBB2 - HA oligomers inhibited the assembly of phosphorylated ERBB2, CD44, ezrin, Hsp90/Cdc37 and p110a into the complex, whereas the total amount of ERBB2 and p85 remained constant - The formed signaling complex is able to exert PI3K/Akt signaling, promoting apoptosis resistance	Ghatak et al. (2005)
		PAR1b	Breast cancer cell line MCF7	Knockdown of total CD44	- Increased levels of HYAL2 promote degradation of HMW-HA to LMW-HA - LMW-HA competes with HMW-HA for CD44 interaction, increases inhibitory phosphorylation of MST1/2 and promotes YAP activation - Knockdown of CD44 resulted in the inverse effect of LMW-HA-CD44 interaction	Ooki et al. (2019)
		EGFR and CD147	Breast cancer cell lines MDA-MB-231 and MCF-7 and immortalized human breast epithelial cell line MCF-10A	Knockdown of total CD44	- Decrease in EGFR activity levels - Lipid raft-associated CD44, CD147 and EGFR activate EGFR and ERK through the CD44-HA signaling enhancement - EGFR-Ras-ERK signaling is known to inhibit apoptosis and promote cell survival - CD44^−^CD147-EGFR axis most likely promotes cell survival through apoptosis inhibition	Grass et al. (2013)
	Ferroptosis	HA-bound iron	Breast and prostate cancer, fibrosarcoma, osteosarcoma cell lines MDA-MB-468, MCF7, LNCaP, HT1080, U2OS and immortalized human breast epithelial cell line HMLER	Knockdown of total CD44	- Decrease in iron uptake - LMW-HA interacts with iron and is able to be internalized in complex with CD44 by endocytosis - CD44-HA-bound iron increases the nuclear iron pool by increasing the levels of nuclear transferrin and reduces the histone mark H3K9me2 in an iron-dependent manner through the upregulation of iron-dependent demethylase PHF8 - Upregulation of genes including CD44 itself in an iron-dependent positive feedback loop with an increasing uptake of CD44-HA bound iron - Currently no research highlighting that iron flux mediated by CD44 significantly contributes to chemoresistance	Müller et al., 2020
	Autophagy	OPN	Pancreatic cancer cell lines PANC-1, MIA PaCa-2, and AsPC-1	-	- OPN knockdown decreases the levels of LC3-II, ALDH1, CD44, and CD133 expression at the protein level - NF-κB, ERK, and STAT3 signaling pathways are activated by OPN - Only NF-κB inhibitor BAY 1170–82 significantly inhibited the OPN-induced LC3-II expression and cell populations with CD44+/CD133+ expression profile	Yang et al. (2015)
CD44v3	Apoptosis	c-Met receptor	Burkitt’s lymphoma cell line Namalwa	Overexpression of CD44v3 (CD44v3-v10)	- c-Met activation through HGF presentation - Activation of c-Met downstream effector molecules ERK1 and ERK2	van der Voort et al. (1999)
		ERBB4	Neuroblastoma and Burkitt’s lymphoma cell lines SH-SY5Y and Namalwa	Overexpression of CD44v3 (CD44v3-v10)	- Enhanced recruitment of an active form of MMP7 and a precursor of HB-EGF by CD44v3-HS side chains - Cleavage of precursor HB-EGF by MMP7 and its presentation to ERBB4 - HB-EGF activation of ERBB4 - Promotion of cell survival through apoptosis inhibition	Yu et al. (2002)
		Vav2 and ERBB2	Ovarian cancer cell line SK-OV-3	-	- CD44v3 ICD was shown to interact with Vav2, a GDP/GTP exchanger for Rac1 - HA increased the rate of Vav2-mediated GDP/GTP exchange reaction compared to untreated cells - HA treatment stimulated ERBB2 activity and caused a significant increase in the amount of CD44v3 complex-associated ERBB2, Grb2 and Vav2 - The formed complex lead to Ras signaling activation and tumor growth promotion	Bourguignon et al. (2001)
		Oct4/Sox2/Nanog	Head and neck cancer stem cells	Cancer stem cells with CD44v3^high^/ALDH1^high^ phenotype	- HA addition caused CD44v3-associated Oct4, Sox2 and Nanog physical complex formation −15–20 min after HA addition, Oct4-Sox2-Nanog accumulation was detected in the nucleus and dispersed in the cytoplasm with HA absence - HA addition caused Oct4, Sox2, or Nanog to bind to the miR-302 cluster promoter region - Knockdown of Oct4, Sox2, or Nanog effectively blocked HA-mediated Oct4-Sox2-Nanog binding to the miR-302 cluster promoter region - downregulation of miR-302a and miR-302b decreased HA-induced anti-apoptotic IAP protein expression, promoting chemosensitivity to cisplatin	Bourguignon et al. (2012)
		-	Bladder cancer cell line HT1376	Knockdown of CD44v3	- Decrease in PI3K, pAKT, pERK, pSTAT3 and Bcl2 protein levels - Arrest of bladder cancer cells in the G0/G1 phase and apoptosis - Combinational treatment with cisplatin and doxorubicin with 4-MU caused a higher decrease in cell viability compared with cisplatin or doxorubicin alone - Combinatorial treatment caused the decrease of PI3K, pAKT, pERK, pSTAT3 and Bcl2 expression at the protein level	Anand et al. (2019)
CD44v6	Apoptosis	FAS receptor	Acute T cell leukemia cell line Jurkat	Overexpression of CD44v6 and CD44s	- FAS-crosslinking antibody treatment had minimal apoptosis inducing effect on CD44v6 cells - CD44 with no variant exons was susceptible to apoptosis - Anti-CD44v6 antibodies (clones VFF18 and BBA13) sensitized Jurkat cells to apoptosis	Mielgo et al. (2006)
		c-Met receptor	Pancreatic adenocarcinoma cell line ASML	Knockdown of CD44v4-v7	- Increase in caspases 9 and 3 cleavage and cytochrome c in the cytoplasm - ASML^wt^ cells demonstrated increased levels of phosphorylated Akt and increased mTOR expression in comparison with CD44v4-v7^kd^ - Addition of c-Met inhibitor SU11274 resulted in the decreased phosphorylation levels of Met and ERK1/2	Jung et al. (2011)
			Human breast cancer cell line T-47D	-	- Upon the addition and interaction of HGF with CD44v6 expressing cells, there was an increase in the number of CD44-HGF-bound dimers compared with cells without HGF addition - Diffusion coefficient in the plasma membrane of such complexes increased by twofold, influenced by the actin cytoskeleton - Increase in lateral diffusion across the plasma membrane of CD44v6-HGF dimeric complexes is favorable for rapid interaction and activation of c-Met, resulting in the formation of a CD44v6-c-Met tertiary complex	Tannoo et al. (2024)
		TG2	Squamous cell carcinoma cell lines SCC-13 and HaCaT	Knockdown of CD44v6	- Decrease in total ERK1/2 levels - TG2 and CD44v6 both form complexes with ERK1/2, as well as with themselves (immunoprecipitation assay) - CD44v6 ICD is necessary for CD44v6/TG2 complex formation - Cancer xenografts display a significant decrease in tumor growth compared to the control, accompanied by reduced CD44v6 and ERK1/2 levels	Chen et al. (2023)
	Autophagy	-	Colon cancer cell lines SW480 and SW620	Overexpression of CD44v6	- Increased levels of BECN1 under treatment by 5-FU - Treatment of control cells and CD44v6-overexpressing cells with 5-FU and an autophagy inhibitor QC resulted in an increase of CD44v6-overexpressing cells sensitivity to the combination of 5-FU and QC - Treatment of CD44v6-overexpressing cells with 5-FU induced higher levels of p-Akt and p-ERK1/2	Lv et al. (2016)
CD44v9	Apoptosis	FAS receptor	Acute T cell leukemia cell line Jurkat	Overexpression of CD44v9	- FAS-crosslinking antibody treatment had minimal apoptosis inducing effect on CD44v9 cells - CD44 with no variant exons was susceptible to apoptosis - Anti-CD44v9 antibody (clone FW11.24) did not restore the potential of cells to undergo apoptosis	Mielgo et al. (2006)
	Ferroptosis	xCT	Gastric and colorectal cell lines MKN28, AGS, KATOIII, HT29 and HCT116	Knockdown of CD44v9 (isoform CD44v8-v10)	- Increase of xCT expression at the mRNA level but decrease of expression at the protein level	Ishimoto et al. (2011)
		OTUB1	Human embryonic kidney cells, neuroblastoma, colorectal, metastatic renal cell, non-small cell lung and bladder cancer cell lines HEK293, SK-N-BE (2)C, U2OS, HCT116, SKRC-42, H1299, T24, UM-UC-3 and SW780	Knockdown of total CD44	- Reduction of co-immunoprecipitated OTUB1 and SLC7A11 - Decrease of SLC7A11 expression and increase in ferroptosis sensitivity in H1299 cells - N-terminal domain of xCT is required for interacting with OTUB1, whereas the C-terminal domain of xCT is critical for binding CD44	Liu et al. (2019)
		MUC1-C	Breast cancer and human embryonic kidney cells MDA-MB-468, MCF-7 and HEK 293T	Transient overexpression of CD44v9 (isoform CD44v8-v10)	- Increase in MUC1-C/xCT complexes - Treatment of MUC1-C^high^ TNBC cell lines with ferroptosis inducer/xCT inhibitor erastin was ineffective in ferroptosis execution - Consequent receptor silencing led to the consecution of erastin-induced ferroptotic cell death, uncovering an additional pathway for ferroptosis modulation	Hasegawa et al. (2016)

**FIGURE 3:**
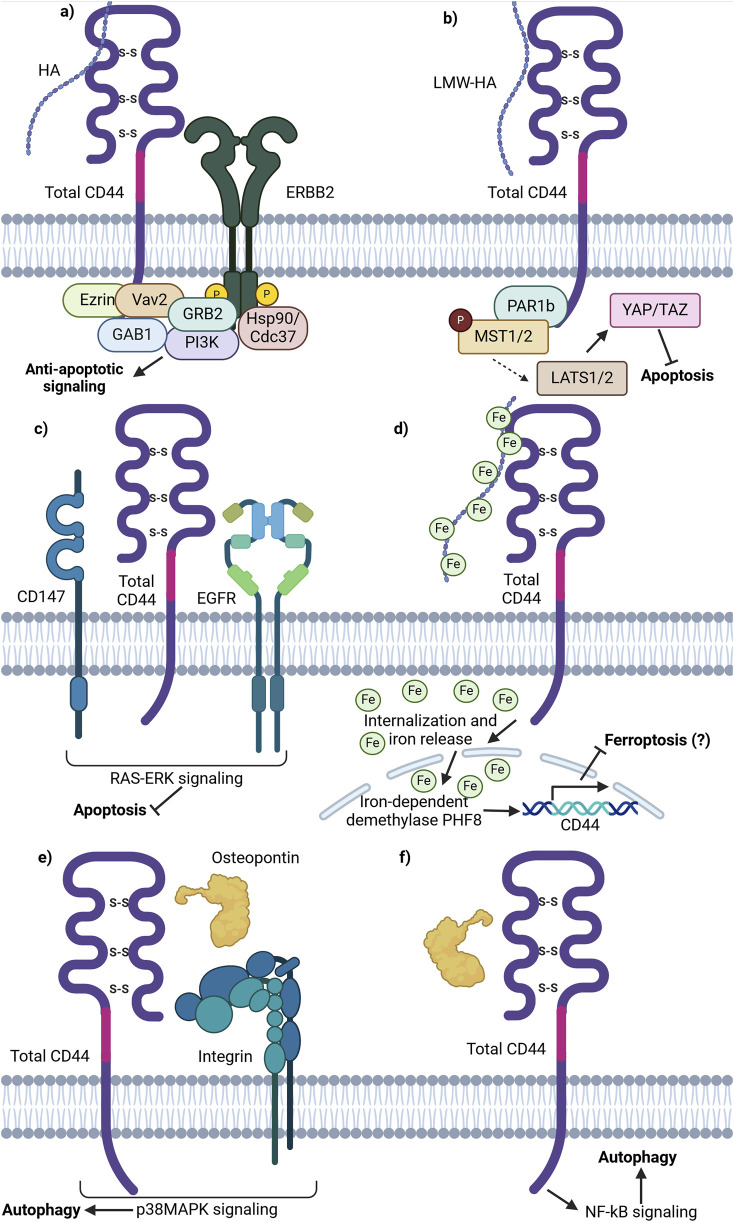
Schematic representation of total CD44 (CD44v and CD44s) involvement in cell death pathways. **(A)** CD44 is able to form an anti-apoptotic complex with ERBB2. CD44 binds ezrin, Vav2 and GAB1 at the ICD. ERBB2 recruits GRB2, Hsp70/Cdc37 and PI3K. The complex formation results in PI3K/Akt anti-apoptotic signaling promotion upon HA stimulation. **(B)** Binding of LMW-HA by CD44 leads to inhibitory phosphorylation of MST1/2 by PAR1b and consequent YAP activation, thereby promoting apoptosis inhibition and cell growth and proliferation activation. **(C)** Lipid raft associated CD44, CD147 and EGFR complex promotes EGFR signaling through the RAS-Erk downstream signaling proteins, conferring anti-apoptotic signaling. **(D)** Total CD44 is involved in the uptake of HA-bound iron, necessary for the function of iron-dependent proteins including nuclear demethylases. Nuclear demethylase PHF8, activated upon iron binding, promotes the expression of CD44 in a positive-feedback loop. **(E)** OPN-CD44-ITG-p38MAPK promotes autophagy. OPN-induced autophagy was inhibited by adding an ITG inhibitor RGD and/or anti-CD44 antibody; p38MAPK inhibitor SB203580 significantly attenuated autophagy by decreasing the expression levels of *ATG4B, BECLIN1/ATG6, BNIP3 and VPS34* (not shown). **(F)** OPN-CD44-NF-κB promotes autophagy. NF-κB inhibitor BAY 1170–82 significantly inhibited the OPN-induced LC3-II expression and cell populations with CD44^+^/CD133^+^ expression profile (Created with BioRender.com).

It was shown that CD44 is able to form a complex with receptor tyrosine-protein kinases 2 (ERBB2) (Ghatak et al., 2005). For instance, HA oligomers, inhibitors of HA interaction with CD44, were shown to prevent the assembly of phosphorylated ERBB2, CD44, ezrin, Hsp90/Cdc37 and p110a into a complex, which is then able to exert ERBB2 associated PI3K/Akt signaling pathway, promoting apoptosis resistance (Ghatak et al., 2005; Figure 3A). Such an interaction demonstrates the involvement of CD44 in growth factor receptor signaling promotion.

Moreover, research on CD44 influence on cell death signaling pathways uncovered its involvement in the Hippo pathway. It was demonstrated that upon HA binding to CD44, CD44 clustering on the cellular membrane is initiated, stimulating the interaction of its intracellular domain with polarity-regulating kinase (PAR1b) (Ooki et al., 2019). Notably, it has been demonstrated that in normal tissues with increasing cell densities, CD44 is activated by high molecular weight (HMW)-HA, thereby initiating PAR1b phosphorylation process of mammalian STE20 like kinase 1 and 2 (MST1/2) and activation of Yes associated protein (YAP). Such a process results in the inhibition of cell growth. Similarly, merlin/neurofibromin-2 (Mer/NF2) also acts as a downstream effector, transferring signals from HA-CD44 activation. CD44 activation by HMW-HA reduces Mer phosphorylation, leading to its activation. Activated Mer has been shown to inhibit PI3K/Akt signaling (Ooki and Hatakeyama, 2020). Overall, in parallel with PAR1b, Mer acts as downstream effector molecules of the HA/CD44/Hippo pathway, modulating its activity. Since the Hippo pathway is an essential survival-associated signaling pathway, its’ inactivation promotes cell proliferation and decreases apoptosis, contributing to tumor initiation, progression and chemoresistance (Figure 3B).

Another pathway utilized by tumor cells to prevent apoptosis is signaling through the CD44/CD147/EGFR complex. On one hand, it was demonstrated that the knockdown of total CD44 leads to decreased EGFR activity levels, pointing to their functional linkage with one another (Grass et al., 2013). On the other hand, CD147 was shown to promote HA synthesis, thereby acting on CD44 activity levels through ligand interactions (Grass et al., 2013). The authors further demonstrated that the CD44/CD147/EGFR complex forms and promotes ERK activation through enhanced CD44-HA signaling in lipid rafts (Grass et al., 2013). While the impact of ERK activation on apoptosis was not directly analyzed, EGFR-Ras-ERK signaling is known to inhibit apoptosis and promote cell survival, thereby implying yet another case of CD44 apoptosis regulation through the interaction with cell membrane receptors (Figure 3C; Ullah et al., 2022).

Apart from apoptosis resistance, total CD44 may be involved in ferroptosis resistance. Müller et al. described a novel mechanism involving hyaluronan-bound iron transfer into the cell via CD44, influencing gene expression regulation (Müller et al., 2020; Figure 3D). The authors demonstrated that LMW-HA bound by iron is internalized intracellularly in complex with CD44 by endocytosis. Similar to transferrin-bound iron, CD44-HA-bound iron is further released into the cytoplasm upon acidification of the endosome and can then be transferred into specific cellular compartments. In particular, CD44-HA-bound iron increases the nuclear iron pool and reduces the histone mark H3K9me2 in an iron-dependent manner (Müller et al., 2020). This leads to the upregulation of genes, including CD44 itself. While no current studies suggest that CD44-mediated iron flux directly contributes to chemoresistance, a potential link may exist. Additionally, the impact of different CD44 isoforms on the CD44-HA-iron feedback loop remains unclear.

Total CD44 is additionally known to regulate autophagy in cancer cells. For instance, autophagy was shown to promote a CD44^high^/CD24^low^ phenotype in breast cancer stem cells (CSCs) – such a cell expression profile was identified in a smaller portion of cells upon knockdown of several autophagy initiating proteins (ATG8/LC3B and ATG12) (Cufí et al., 2011). Moreover, the treatment of CD44^high^/CD24^low^ cells with an autophagy inhibitor chloroquine (CQ) was shown to shift the cancer cell phenotype to the opposite (CD44^low^/CD24^high^) (Cufí et al., 2011). Additionally, the CD44 ligand osteopontin (OPN) has been implicated in autophagy induction. In vascular smooth muscle cells (SMCs), OPN was shown to trigger autophagosome formation (Kariya and Kariya, 2022). This process was inhibited by an integrin (ITG) inhibitor (RGD peptide) or anti-CD44 antibody, highlighting the importance of CD44 and RDG motif-containing integrins in autophagy initiation (Figure 3E). In another study, OPN activation was shown to stimulate NF-κB, ERK, and STAT3 signaling pathways (Figure 3F; Zheng et al., 2012). Thus, literature suggests that total CD44 and its ligands are linked with autophagy initiation and the stemness properties of tumor cells, thereby promoting resistance to administered therapy.

Overall, the mechanisms responsible for total CD44 mediation of cell death pathways are diverse. They include the influence of apoptosis resistance through ERBB2 complex, CD147 and CD44 interaction with LMW-HA. Currently, no research highlights that iron flux mediated by CD44 significantly contributes to chemoresistance, but there may be an association. Whether different CD44 isoforms alter their expression patterns within the CD44-HA bound iron positive feedback loop remains unclear. Further studies are required to clarify these possible associations and their relationship with chemoresistance. Lastly, total CD44 was shown to promote autophagy and chemoresistance in several cancers through OPN-CD44-ITG-p38MAPK and OPN-CD44-NF-κB signaling. Thus, targeting both cell death pathways and total CD44 signaling may offer a promising approach to overcoming chemoresistance in various cancers. However, it should be mentioned that additional research comparing the effect of standard and variant isoforms in mediating cell death signaling may be necessary for further precision in targeting chemoresistant cells.

### 5.3 Total CD44 therapeutic strategies and clinical implications

Several approaches targeting total CD44 (including variant and standard isoforms) for chemotherapy resistance alleviation have been developed and analyzed *in vivo* (Table 4). Some approaches for CD44-exerted chemoresistance treatment entail using CD44-targeting gene therapies, delivery systems and cell therapies. Regarding gene therapy strategies, complementary DNA (cDNA) vaccine to CD44 variant isoforms, but not CD44s, demonstrated decreased tumor growth, aggressiveness and metastasis in breast cancer xenografts (Wallach-Dayan et al., 2008). It was identified that the cDNA vaccine induced the production of antibodies recognizing CD44 variant isoforms (Wallach-Dayan et al., 2008). In addition, several studies revealed the beneficial effect of CD44-targeting with micro-RNA (miRNA). MiR-199a overexpression in CD44^+^ cancer-initiating cells (CICs) in ovarian cancer xenografts significantly decreased tumor volume (Cheng et al., 2012). Additionally, it was identified that a more significant percentage of apoptotic cells is formed with miR-199a overexpression. Similar effects were observed with the overexpression of miR-24a and miR-34a in gastric and esophageal cancer xenografts (Jang et al., 2016; Zuo et al., 2018). Overall, the data surrounding gene therapy approaches seems promising regarding cancer patient treatment, but they should be cautiously studied since they can carry the risk of off-target effects.

**TABLE 4:** Summary of *in vivo* studies using CD44-targeted therapy.

Targeted CD44 exon	Approach	Expression in cancer	Therapeutic substance	Response *in vivo*	Source
Total CD44	Gene therapies	Ovarian cancer xenografts of CICs	miR-199a	- Decrease in tumor volume - Weak cell proliferation-related protein Ki67 staining - Detection of greater amounts of apoptotic cells	Cheng et al. (2012)
		Gastric cancer xenografts of cell line MKN-74	miR-24a	- Nanovesicles containing miR-34a decreased tumor volume by more than 50% - Greater amount of detected apoptotic cells	Jang et al. (2016)
		Esophageal squamous cell carcinoma xenografts of cell lines ECA109 and TE-13	miR-34a	- Tumors with miR-34a knockdown demonstrated an increase in tumor weight and size - MiR-34a overexpression exerted the opposite effect - MiR-34a knockdown increased CD44 expression levels, whereas miR-34a overexpression led to CD44 decrease in expression levels	Zuo et al. (2018)
	Chemotherapy delivery systems	Breast cancer xenografts of cell line 4T1	pH-sensitive micelles loaded with doxorubicin and incorporated HA in its structure	- Decrease in tumor growth rate and apoptosis induction in comparison with doxorubicin alone	Yang et al. (2021)
	Cell therapies	Hepatocellular carcinoma xenografts of CD44^+^ cell lines Hep3B2, MHCC97H and SMMC-7721 and CD44^−^ cell lines PLC8024 and HepG2	CAR-T cells	- CD44^+^ tumors richly infiltrated by CD44-CAR T cells - CD44^−^ tumors had seldom infiltration with CD44-CAR T cells - Decreased tumor volumes of CD44^+^ xenografts treated with CD44-CAR T cells - Tumor volume alteration of CD44^−^ tumors was nonsignificant with CD44-CAR T cells	Wang et al. (2016a)
CD44v	Gene therapies	Breast cancer xenografts of mouse cell line DA3	CD44v cDNA vaccine	- Decrease of tumor mass and aggressiveness	Wallach-Dayan et al. (2008)
	Chemotherapy delivery systems	Oral cancer xenografts of cell line HSC2	Micelles incorporating cisplatin	- Reduced overall tumor growth rate and volume - Cisplatin alone demonstrated a short-term suppressive effect on tumor growth rate	Wang et al. (2016b)
CD44v6	Antibodies/peptides	Pancreatic cancer xenografts of human cancer cells L3.6 pL and JoPaca-1	Hv6pep	- Tumor shrinkage - The v6 peptide was more efficient than MET and/or VEGFR-2 inhibitors crizotinib and pazopanib in decreasing tumor growth	Matzke-Ogi et al. (2016)
	Cell therapies	Ovarian cancer xenografts of cell line IGROV-1	CAR-T cells	- Antitumor effects and enhanced survival (median survival of 27 and 37 days in CD19-CAR and CD44v6-CAR treated groups of mice) - CD44v6-CAR T cells inhibited tumor growth and prolonged overall survival (median survival of 21.5 and 37 days in CD19-CAR and CD44v6-CAR treated groups of mice)	Porcellini et al. (2020)
CD44v8 (CD44v8-v10)	Antibodies/peptides	Uterine cervix and larynx xenografts of cell lines Me180 and HSC-3	GV5	- Inhibition of tumor growth	Masuko et al. (2012)
CD44v9	Pharmacological inhibitors	Hepatocellular carcinoma xenografts of cell line HAK-1B	SAS; cisplatin	- Decrease in tumor size by treatment with cisplatin alone and cisplatin with SAS	Wada et al. (2018)
		Metastatic bladder cancer xenografts of cell line MBT-2V	SAS; cisplatin	- Combination treatment decreased the number of tumor nodules in comparison with control, cisplatin alone and SAS alone - CD44v9 density was significantly lower in SAS and cisplatin combination therapy group in comparison with control, cisplatin alone and SAS alone	Ogihar et al. (2019)

Moreover, CD44 variant isoforms can be helpful for tumor cell targeting (cell therapies) and chemotherapy delivery due to their overexpression in tumor tissue. Anti-CD44 variant isoforms micelles, incorporating cisplatin, have shown success in decreasing the tumor volume of oral cancer xenografts by more than 50% (Wang et al. (2016a)). In addition, CD44-targeted and pH-sensitive doxorubicin-loaded micelles have exerted selective cytotoxic effects in breast cancer xenografts, resulting in apoptosis induction (Yang et al., 2021). A recently developed method for tumor cell targeting is therapy with chimeric antigen receptor (CAR) T cells, successfully applied for CD44^+^ tumor cell elimination. For instance, CD44^+^ hepatocellular carcinoma xenografts treated with CD44-CAR T cells demonstrated a significant decrease in tumor volume in comparison with mock T and normal T cells; CD44 tumor volumes were unchanged in comparison with CD44-CAR T, mock T and normal T cells (Wang et al., 2020).

Even though preclinical research data suggest that total CD44 play a significant role in the chemoresistance of cancer patients, there is a limited amount of conducted clinical studies regarding total CD44 targeted therapies (Table 5). They include therapeutic approaches such as using antibodies/peptides or pharmacological inhibitors.

**TABLE 5:** Clinical trials aimed at targeting CD44 for overcoming chemoresistance. Retrieved from clinicaltrials.org and umin.ac.jp/ctr.

Targeted CD44 exon	Approach	Therapeutic substance	Cancer type	Phase and status	Results	Study number
Total CD44	Antibodies/peptides	RO5429083 (RG7356)	metastatic and/or locally advanced CD44-expressing malignant solid tumors	Phase I completed in 2014	Acceptable safety profile, clinical efficacy is modest (best response was stable disease observed at 8 weeks in 21% of patients)	NCT01358903
		A6	Ovarian cancer	Phase II completed in 2006	50% of patients with asymptomatic biochemical recurrence experienced periods of stable disease for at least 4 cycles	NCT00083928
	Pharmacological inhibitors	GSK1120212 (trametinib)	Oral cavity squamous cell carcinoma	Phase II completed in 2015	Reduction of pERK1/2 and CD44 in 33% of patients, reduction of tumor volume in 65% of patients, tumor downstaging in 53% of patients	NCT01553851
CD44v6	Antibodies/peptides	Bivatuzumab mertansine	Recurrent or metastatic breast cancer	Phase I terminated in 2005	Targets CD44v6, but skin toxicity was detected due to strong expression of CD44v6 in skin keratinocytes. Clinical development discontinued	NCT02254031
			Advanced head and neck squamous cell carcinoma	Phase I completed in 2005	One patient developed stable disease during treatment phase. Main toxicity directed against the skin, one fatal drug-related adverse event occurrence. Clinical development discontinued	NCT02254018
	Pharmacological inhibitors	AMC303	Multiple solid cancers	Phase I completed 2021	Demonstrated to be well-tolerated by patients. Part 2 is designed to test anti-tumor activity	NCT03009214
	Cell therapies	CAR T cells	Breast cancer	Phase I/II unknown status	Unknown status (the study passed its completion date, but the status has not been verified)	NCT04430595
			Multiple cancers	Phase I/II unknown status	Unknown status (the study passed its completion date, but the status has not been verified)	NCT04427449
			Acute myeloid leukemia and multiple myeloma	Phase I/II terminated	Terminated due to inability to close the study in a clinically relevant time frame	NCT04097301
CD44v9	Pharmacological inhibitors	SAS	Advanced gastric cancer	Phase I completed in 2016	Decrease of CD44v positive cells by more than 10% in 50% of patients. Significant reduction of GSH levels in 70% of patients. Best response to therapy was stable disease	EPOC1205
			Gastric cancer refractory to cisplatin	Phase I completed in 2017	One patient achieved stable disease for more than 4 months. Three other patients showed stable disease for limited duration	EPOC1407

**FIGURE 4:**
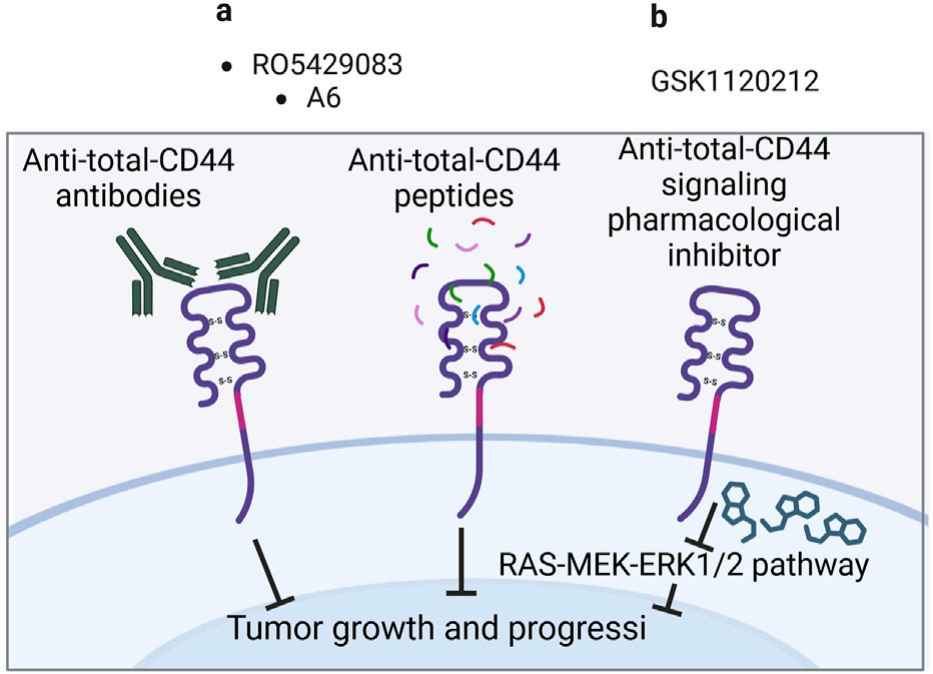
Clinical trials aimed at targeting total CD44 (Created with bioRender.com). **(A)** Antibodies/peptides. **(B)** Pharmacological inhibitors.

Anti-total-CD44 targeting approaches with modest/insufficient effect have been noted (NCT01358903): an anti-CD44 antibody RO5429083 (alternatively named RG7356) was tested in metastatic and/or locally advanced CD44-expressing malignant solid tumors (Menke-van der Houven van Oordt et al., 2016). The developed antibodies demonstrated an acceptable safety profile, however, clinical efficacy was identified to be modest–the best response to chemotherapy was stabilization of disease progression identified in 21% of patients. Some promising clinical trials have also been conducted (NCT00083928 and NCT01553851). A6 peptide, a short amino acid sequence targeting the HA-binding domain of CD44, performed well in increasing the time to progression of patients with ovarian cancer: half of the patients with asymptomatic biochemical recurrence experienced periods of stable disease for at least 4 cycles of administered A6 peptide therapy (Ghamande et al., 2008). Moreover, trametinib (conversely named GSK1120212) was shown to decrease Ras/MEK/ERK pathway activation and CD44 expression, resulting in a beneficial clinical response in patients (the effect was seen in 11 out of 17 patients), a decrease in tumor volume and tumor downstaging (Figure 4; Uppaluri et al., 2017).

## 6 CD44 variant exon 3 and chemoresistance

### 6.1 Effect of CD44 variant exon v3 expression on chemotherapy treatment outcome/tumor response to chemotherapy in patients/animal studies

CD44v3 seems to be the least studied variant exon out of the group of clinically relevant exons (Table 6). Concerning the correlation of expression levels with clinical outcomes of patients, it was demonstrated that high protein expression levels of CD44v3 exert chemoradiotherapy resistance in a study with nasopharyngeal patients (Sagawa et al., 2016). There seem to be no studies examining the association between CD44v3-containing isoforms overexpression/knockdown and chemoresistance *in vivo*. Nevertheless, the biological effect of CD44v3 has been studied *in vitro.*

**TABLE 6:** The association of CD44 variant/only constant exon expression levels with clinical outcomes of cancer patients.

CD44 variant/only constant exons	Expression in cancer	Administered chemotherapy	Correlation with patient’s outcome	Detection method	Source
CD44v3	Nasopharyngeal cancer	Carboplatin-based chemoradiotherapy	Poor DSS	IHC	Sagawa et al. (2016)
CD44v4	Nasopharyngeal cancer	Carboplatin-based chemoradiotherapy	No correlation with DSS	IHC	Sagawa et al. (2016)
CD44v5	Nasopharyngeal cancer	Carboplatin-based chemoradiotherapy	No correlation with DSS	IHC	Sagawa et al. (2016)
CD44v6	Nasopharyngeal cancer	Carboplatin-based chemoradiotherapy	Poor DSS	IHC	Sagawa et al. (2016)
	Esophageal squamous cell carcinoma	Cisplatin, 5-FU and adriamycin/docetaxel-based chemotherapy	Poor OS in combination with c-Met expression	IHC	Hara et al. (2019)
	Tongue cancer	Cisplatin or carboplatin-based chemotherapy	Poor DSS	IHC	Yanamoto et al. (2014)
	Colorectal cancer	Surgery and chemotherapy (not specified)	Tumor recurrence	IHC	Lv et al. (2016)
CD44v7	Nasopharyngeal cancer	Carboplatin-based chemoradiotherapy	Poor DSS	IHC	Sagawa et al. (2016)
CD44v9	Upper tract urothelial carcinoma	Surgery and cisplatin-based chemotherapy	Poor RFS and DSS	IHC	Hagiwara et al. (2016)
	Metastatic and/or recurrent bladder cancer	Cisplatin-based chemotherapy	Poor DSS	IHC	Hagiwara et al. (2016)
	Non-chemoradioselected advanced head and neck cancer	Chemoradiotherapy (not specified)	Poor DSS	IHC	Aso et al. (2015)
CD44v10	Oral cancer with local recurrence	Chemoradiotherapy (not specified)	Tumor recurrence	IHC, PCR	Kashyap et al. (2018)
CD44s	Oral cancer with local recurrence	Chemoradiotherapy (not specified)	No correlation with tumor recurrence	IHC	Kashyap et al. (2018)
	Advanced-stage ovarian carcinoma	Surgery and cisplatin-based chemotherapy	No correlation with OS and DFS No correlation with chemotherapy response	IHC	Zhang et al. (2013)
	Ovarian carcinoma	Surgery and cisplatin-based chemotherapy	Increased OS	IHC	Ross et al. (2001)

### 6.2 CD44 variant exon v3 biological functions in relation to cell death pathways

The biological effects exerted by variant exons of CD44 with significant clinical association concerning cell death pathways are summarized in Table 3. They will be covered in detail in the upcoming sections of our review.

The study of a particular exon’s functional role is quite tricky. Such methods as overexpression/knockdown of particular CD44 isoforms, which typically contain more than one variant exon, are widely applied. Indeed, we are not the first to review the involvement of CD44 variant exons in chemoresistance induction through apoptosis regulation (Wang et al., 2018). However, Wang et al. primarily focus on variant exon v6. In our review, we outline what is known to date about all clinically relevant variant exons regarding cell death mechanisms, including apoptosis, ferroptosis and autophagy.

**FIGURE 5:**
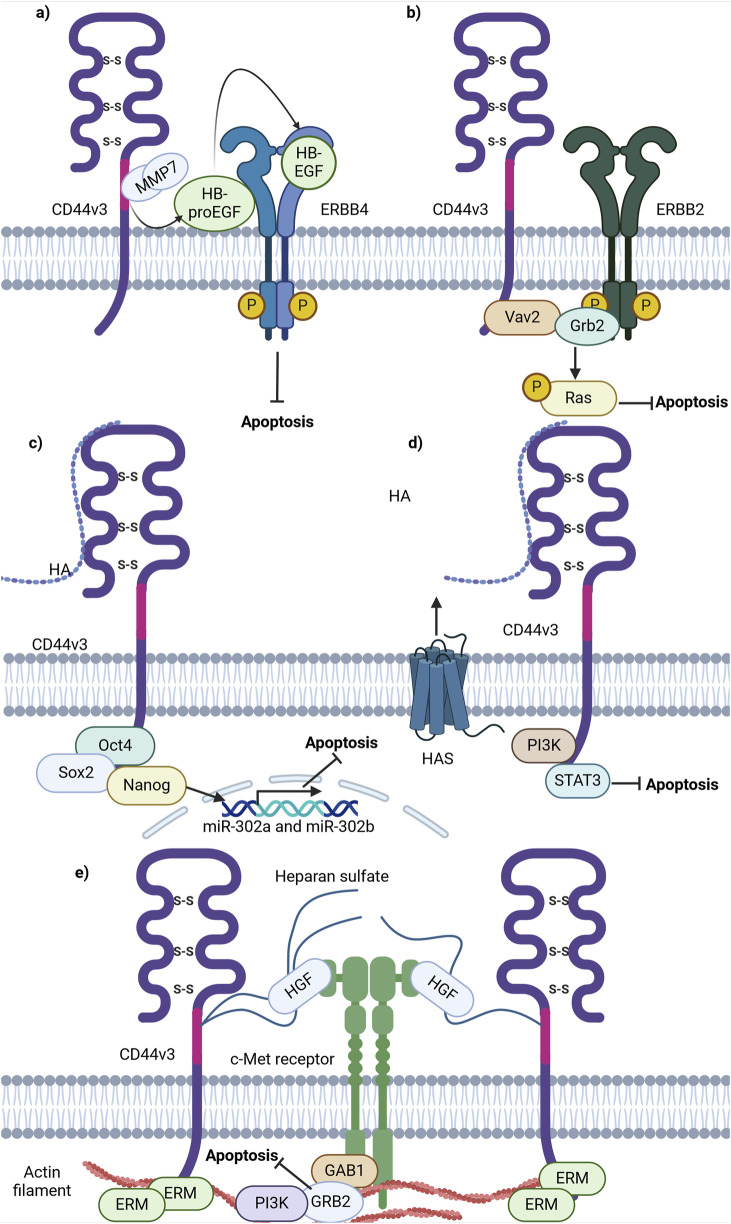
Schematic representation of CD44 variant exon v3 interaction with cellular receptors/intracellular proteins involved in cell death signaling. **(A)** CD44v3 containing isoforms recruit MMP7 and inactive HB-EGF, leading to consequent cleavage, activation and presentation of HB-EGF to ERBB4, thereby promoting anti-apoptotic signaling. **(B)** CD44v3 containing isoforms form a complex with ERBB2, Vav2 and Grb2 and promote anti-apoptotic Ras-MAPK signaling. **(C)** The ICD of CD44v3 containing isoforms promotes the formation of Oct4, Sox2 and Nanog complex and its consequent shift to the nucleus. The intranuclear Oct4/Sox2/Nanog complex promotes the expression of miR-302a and miR-302b, promoting anti-apoptotic signaling. **(D)** CD44v3 promotes antiapoptotic signaling through PI3K/STAT3 pathway. **(E)** CD44v6 containing isoforms activate c-Met anti-apoptotic signaling: upon binding of HGF, CD44v6 form dimeric complexes (not shown in figure) that are able to laterally diffuse through the plasma membrane for rapid interaction with c-Met with the formation of an active trimeric complex (Created with BioRender.com).

Firstly, CD44 has been shown to bind some metalloproteinases (MMPs), a family of endopeptidases that hydrolyze components of the extracellular matrix (Hassn Mesrati et al., 2021). Specifically, CD44v3 containing isoforms were identified to recruit the active form of MMP-7 and the precursor of HB-endothelial growth factor (HB-EGF) at its extracellular domain. This is possible due to HB-EGF binding of HS-rich sites in variant exon v3 of CD44 (Yu et al., 2002). Such an interaction allows the presentation of mature HB-EGF to membrane receptors, such as ERBB4, and thus the promotion of cell survival (Figure 5A; Yu et al., 2002). The mechanism through which CD44 forms complexes at the cellular membrane with additional receptors is quite often utilized by cancer cells and seems to be successful in the promotion of cell death resistance.

Moreover, CD44v3-containing isoforms were shown to promote ovarian tumor growth through ERBB2 signaling. It was identified that the ICD of CD44v3-containing isoforms interacts with adaptor proteins Vav2 and growth factor receptor bound protein 2 (Grb2) (Bourguignon et al., 2001), thus promoting coupling of ERBB2 and CD44v3-containing isoforms to Ras and the promotion of its signaling (Bourguignon et al., 2001; Figure 5B). This interaction is similar to that of total CD44 and ERBB2, but the mechanisms of apoptosis promotion vary. This points to a nonuniform response of tumor cells from different cancers upon interaction of CD44 with the same receptor.

Additionally, CD44v3 was shown to promote apoptosis resistance through epigenetic regulatory mechanisms. For instance, in CSCs with a CD44v3^high^/ALDH1^high^ phenotype, master transcriptional factors Oct4, Sox2 and Nanog were detected to be overexpressed at the mRNA and protein levels (Bourguignon et al., 2012). Interestingly, upon activation of CD44v3-containing isoforms by HA in such cells, CD44v3-associated Oct4, Sox2 and Nanog physical complex formation and dislocation of Oct4/Sox2/Nanog to the nucleus was observed (Bourguignon et al., 2012). Upon nuclear localization, Oct4/Sox2/Nanog was identified to bind the miR-302 cluster promoter region, enhancing the production of miR-302a and miR-302b expression. SiRNA-mediated knockdown of these factors blocked HA-mediated Oct4/Sox2/Nanog binding to the miR-302 cluster promoter and decreased HA-induced antiapoptotic IAP protein expression, promoting chemosensitivity to cisplatin (Bourguignon et al., 2012). Thus, these finding indicate the ability of CD44v3 to utilize regulatory mechanisms not only at the protein level, but also epigenetically.

Further investigations into the role of CD44v3 in apoptosis regulation unveiled that the knockdown of CD44v3-containing isoforms mediates the reduction of PI3K, pAKT, pERK, pSTAT3 and Bcl2 protein levels, triggering apoptotic signaling and G0/G1 phase arrest in bladder cancer cells (Anand et al., 2019). Combinational treatment of cisplatin and doxorubicin with 4-methylumbelliferone (4-MU), an HA synthesis inhibitor, significantly decreased cell viability compared to cisplatin or doxorubicin alone, suggesting that the HA/CD44v3 axis is of importance in apoptotic signaling regulation (Figure 5D; Anand et al., 2019).

As mentioned previously, CD44v3 encodes a heparan sulphate (HS) side-chain attachment motif, allowing HS-bound CD44v3 to bind growth factors including hepatocyte growth factor (HGF) and to present them to a number of cell membrane receptors including the mesenchymal epithelial transition factor receptor (c-Met) (van der Voort et al., 1999). A significant pathway of c-Met signaling is the PI3K/Akt signaling axis primarily responsible for the cell survival response: the p85 subunit of PI3K can bind either directly to c-Met or indirectly through GAB1, which in turn promotes anti-apoptotic signaling through AKT (Xiao et al., 2001). Thus, these findings underline the importance of HS motifs and their utilization by CD44v3-containing isoforms to promote antiapoptotic signaling.

Overall, CD44v3-containing isoforms contribute to apoptosis resistance through several mechanisms, including the c-Met and ERBB2 receptors, epigenetic regulation employing miR-302 cluster upregulation and MMPs. Further research on these pathways will facilitate the development of targeted therapies to overcome chemoresistance and improve cancer treatment outcomes.

### 6.3 CD44 variant exon v3 therapeutic strategies and clinical implications

Despite the relatively large number of molecular mechanisms of resistance in which CD44v3 partakes, there seem to be practically no antibodies or clinical trials aimed at targeting CD44v3. A novel monoclonal antibody, C44Mab-6, was recently established (Suzuki et al., 2023). One advantage of such antibodies is the area of recognition of CD44v3-containing isoforms. For instance, compared with previously established anti-CD44v3 antibodies (clone 3G5), C44Mab-6 recognizes a peptide sequence excluding an HS-modified sequence in the CD44 variant-3-encoded region, indicating that C44Mab-6 antibodies recognition of CD44v3 is not influenced by HS modifications (Suzuki et al., 2023). This may be useful for CD44v3 targeting, increasing the selectivity of such antibodies. The authors mention that further testing of C44Mab-6 *in vivo* is reckoned.

Apart from the development of the antibodies mentioned above, it does not appear that other antibodies have been synthesized and tested in clinical settings. Thus, further research is necessary to determine the relevance of CD44v3 inhibition, considering the small number of studies regarding the analysis of its clinical relevance.

## 7 CD44 variant exon v6 and chemoresistance

### 7.1 Effect of CD44 variant exon v6 expression on chemotherapy treatment outcome and survival of patients and tumor response to chemotherapy in animal studies

One of the most widely and thoroughly studied variant exons concerning the clinical outcome of patients and biological effect is CD44v6 (Table 6). Several studies unveiled the prognostic significance of CD44v6 in nasopharyngeal, esophageal (in combination with c-Met expression) and tongue cancer (Hara et al., 2019; Sagawa et al., 2016; Yanamoto et al., 2014). High protein expression levels of CD44v6 significantly correlated with poor disease-specific survival (DSS) and OS of patients treated with cisplatin/carboplatin-based chemotherapy, as well as with tumor recurrence after chemotherapy treatment in colorectal cancer patients (Lv et al., 2016).

In addition, the association between CD44v6 overexpression/knockdown and chemoresistance *in vivo* has been studied (Table 2). Research indicates that CD44v6^kd^ in prostate and colon cancer xenografts demonstrated a significant decrease in tumor growth and practically complete impalpability by the end of docetaxel administration and in folinic acid, 5-FU and oxaliplatin (FOLFOX)-resistant cells (Ni et al., 2020; Ghatak et al., 2021).

Overall, the implemented literature analysis, although not comprehensive, pointed to a relationship between variant exon CD44v6 high expression levels and worse chemotherapy treatment outcomes in cancer patients. Cancer xenografts confirmed the negative relationship of CD44v6 expression level with worse chemotherapy efficacy *in vivo*. The results demonstrate that CD44v6 may be involved in chemotherapy resistance, and further research into its molecular mechanisms is needed.

### 7.2 CD44 variant exon v6 biological functions in relation to cell death pathways

The ongoing investigation into the role of CD44 variant exon v6 in cellular death pathways across various cancer types may not yet provide a comprehensive understanding for all cancers. To date, CD44v6 is the most studied variant exon out of the other clinically variant exons. It has been shown to influence apoptosis and autophagy.

**FIGURE 6:**
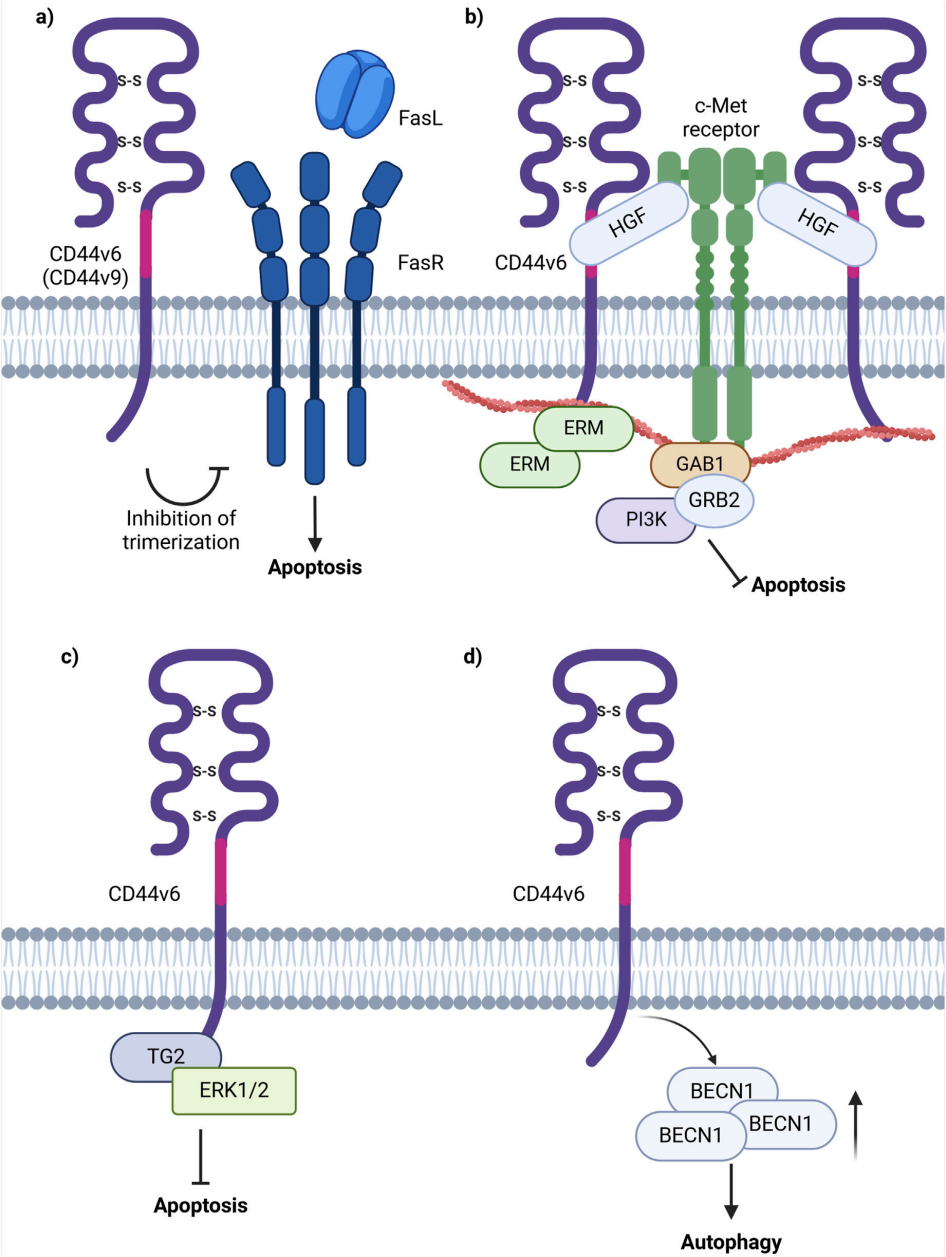
Schematic representation of CD44 variant exon v6 interaction with cellular receptors/intracellular proteins involved in cell death signaling. **(A)** The interference of CD44v6 containing isoforms in apoptosis induction through the extrinsic pathway possibly by blocking the trimerization of FASR, necessary for FASL interaction. **(B)** CD44v6 containing isoforms activate c-Met anti-apoptotic signaling: upon binding of HGF, CD44v6 form dimeric complexes (not shown in figure) that are able to laterally diffuse through the plasma membrane for rapid interaction with c-Met with the formation of an active trimeric complex. **(C)** CD44v6 ICD forms a complex with TG2 and ERK1/2 promoting anti-apoptotic signaling. **(D)** CD44v6 containing isoforms stimulate autophagy induction through increasing the levels of BECN1, one of the key players in autophagic vesicle extension (Created with BioRender.com).

One of the pioneering studies on CD44v6 involvement in apoptosis revealed that CD44 exon v6 containing isoforms exhibit antiapoptotic properties through colocalizing, interacting with and blocking FAS trimerization (Figure 6A; Mielgo et al., 2006). Notably, cells, constitutively expressing FASR and being sensitive to apoptosis with the addition of FASL or FAS-crosslinking antibodies, become resistant to apoptosis with the transfection of CD44 variant isoforms. It was demonstrated that blocking the CD44v6 exon with anti-CD44v6 antibodies (clone VFF18) in cancer cells results in their sensitization to apoptosis, thereby underscoring CD44v6’s role in the promotion of antiapoptotic signaling. The authors further propose that CD44 variant isoforms may interact with the pre-ligand assembly domain of FASR and prevent its trimerization, which is necessary for FASL binding (Mielgo et al., 2006). Thus, CD44v6-containing isoforms employ regulatory mechanisms acting directly on the extrinsic apoptotic signaling pathway.

Moreover, research indicates that CD44v6 interacts with the mesenchymal epithelial transition factor receptor c-Met and inhibits apoptosis through two primary mechanisms: 1) presentation of HGF to c-Met or 2) by direct interaction of c-Met or HGF with the variant exon domain of CD44v6 (Figure 6B). Based on current studies, the function of CD44v6 in c-Met activation is of dual nature. Firstly, the ectodomain of CD44v6-containing isoforms is necessary for c-Met activation through the binding and presentation of HGF. Secondly, the cytoplasmic domain of CD44v6-containing isoforms is linked to ERM proteins and the cytoskeleton and is required for RAS activation (Hasenauer et al., 2013). Overall, CD44v6-c-Met interaction promotes apoptosis through PI3K/Akt and mammalian target of rapamycin (mTOR) pathways (Jung et al., 2011). Notably, studies indicate that upon the addition and interaction of HGF with CD44v6 expressing cancer cells, there is an increase in the number of CD44-HGF-bound dimers and in the diffusion coefficient in the plasma membrane of such complexes (Tannoo et al., 2024). The latter process is favorable for rapid interaction with and activation of c-Met, resulting in the formation of a CD44v6-c-Met tertiary complex (Tannoo et al., 2024). Therefore, tumor cells expressing CD44v6-containing isoforms utilize variant exon v6 for binding of growth factors and their presentation to cell membrane receptors (or even directly interacting with them), promoting anti-apoptotic signaling initiation.

Moreover, the formation of a CD44v6/transglutaminase 2 (TG2)/ERK1/2 protein complex was shown to promote cancer cell growth and resistance to apoptosis (Chen et al., 2023). It was identified that TG2 knockdown leads to decreased levels of CD44v6 and reduced ERK1/2 activity and expression (Chen et al., 2023). Furthermore, TG2 and CD44v6 were found to form complexes with ERK1/2, as well as with each other. In the interaction involving TG2 and CD44v6-containing isoforms, the cytoplasmic domain of CD44 plays a critical role in maintaining the stability of the complex (Chen et al., 2023). Importantly, CD44v6-knockdown xenografts displayed a significant decrease in tumor growth, accompanied by reduced CD44v6 and ERK1/2 levels, thereby utilizing yet another cooperative mechanism with binding partners for anti-apoptotic signaling promotion (Figure 6C; Chen et al., 2023).

Furthermore, overexpression of CD44 isoforms containing CD44v6 (CD44v6-overexpressing cells) demonstrated an increase in resistance to 5-FU in colorectal cancer cells (Lv et al., 2016). An increase in BECN1 expression under treatment by 5-FU in CD44v6-overexpressing cells was identified, and treatment of CD44v6-overexpressing cells with 5-FU and an autophagy inhibitor quinacrine (QC) resulted in an increase of CD44v6-overexpressing cell’s sensitivity to the combination of 5-FU and QC, indicating that CD44v6 promotes autophagy resistance. In another study, the knockdown of CD44v6 containing isoforms in colorectal cancer cells resulted in a significant decrease in *BECN1* levels, further validating the results obtained in the previously mentioned study (Wang et al., 2019; Figure 6D).

Thus, the role of CD44v6 in apoptosis resistance, autophagy promotion, and consequent chemoresistance attenuation is complex, and it is involved in modulating several signaling pathways and interactions. CD44v6-containing isoforms contribute to apoptosis resistance through mechanisms involved in both direct apoptosis regulation via FASR and indirect apoptosis influence through the c-Met receptor. Additionally, CD44v6-containing isoforms were shown to promote autophagy in several cancers. These interactions underline the complexity of CD44’s functions responsible for cancer progression and resistance to treatment. The development of targeted therapeutic drugs for overcoming chemoresistance greatly depends on understanding CD44 variant exon-containing isoforms manipulation of cell survival signaling pathways.

### 7.3 CD44 variant exon v6 therapeutic strategies and clinical implications

Given the seemingly promising role of CD44v6-containing isoforms in cancer chemoresistance demonstrated in preclinical studies, several approaches targeting CD44v6 for chemotherapy resistance alleviation have been developed and analyzed *in vivo* (Table 4). These strategies aim to disrupt CD44-mediated signaling, enhance cell death induction and, as a result, overcome chemoresistance.

Some approaches for CD44v6-exerted chemoresistance treatment entail the use of anti-CD44v6 antibodies/peptides or pharmacological inhibitors. For instance, CD44v6 targeting peptides Hv6pep, developed with the inclusion of 3 amino acids necessary for v6 interaction with coreceptors, induced a significant tumor growth reduction upon xenograft injection. Notably, the anti-CD44v6 peptide was more effective in tumor growth inhibition than inhibitors of CD44v6 interactions with coreceptors c-Met and vascular endothelial growth factor receptor 2 (VEGFR-2) (Matzke-Ogi et al., 2016). A recently developed method for tumor cell targeting is therapy with chimeric antigen receptor (CAR) T cells, successfully applied for CD44v6 tumor cell elimination. For instance, the developed CD44v6-CAR T cells demonstrated antitumor effects and significantly prolonged survival (median survival of 27 and 37 days in CD19-CAR and CD44v6-CAR treated groups) of mice with ovarian cancer tumors (Porcellini et al., 2020). Thus, CD44v6-expressing isoforms are helpful for selective and direct tumor cell annihilation and precise drug delivery to chemoresistant cells.

Even though preclinical research data and several case reports suggest that CD44v6-containing isoforms play a significant role in the chemoresistance of cancer patients, there is a limited amount of conducted clinical studies regarding CD44v6-targeted therapies (Table 5). Cases of insufficiently effective anti-CD44v6 targeting approaches have been noted (NCT01358903, NCT01358903 and NCT02254018).

**FIGURE 7:**
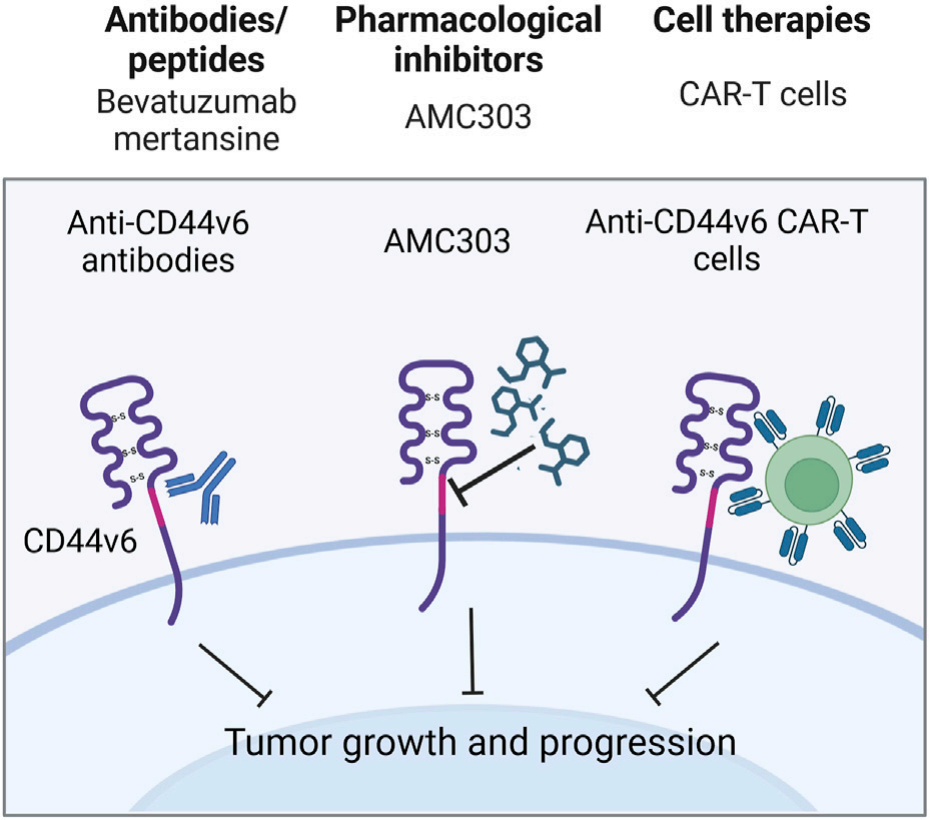
Clinical trials aimed at targeting CD44v6 containing isoforms (Created with bioRender.com).

An anti-CD44v6 antibody, bivatuzumab, in combination with mertansine, was tested in studies targeting recurrent or metastatic breast cancer and advanced head and neck carcinoma (Figure 7; Koppe et al., 2004; Riechelmann et al., 2008; Ishimoto et al., 2011). In the cases of both metastatic breast cancer and advanced head and neck carcinoma, bivatuzumab was shown to target CD44v6, although not only on tumorous tissue, but additionally on skin keratinocytes - cells strongly expressing CD44v6. In the head and neck carcinoma study, one patient developed stable disease during the treatment phase, but a patient’s drug-related severe skin toxicity caused the study termination due to their death. Lastly, phase I/II clinical studies aimed at targeting CD44v6 with CAR T cells (NCT04430595, NCT04427449 and NCT04097301) and with pharmacological inhibitor AMC303 (NCT03009214) have demonstrated exemplary safety, and further analysis with respect to clinical effect is expected (Figure 7).

## 8 CD44 variant exon 9 and chemoresistance

### 8.1 Effect of CD44 variant exon v9 expression on chemotherapy treatment outcome/tumor response to chemotherapy in patients/animal studies

Another frequently studied variant exon is CD44v9, which was shown to be associated with poor RFS and DSS of the upper urinary tract and urothelial cancer patients undergoing cisplatin-based chemotherapy, as well as non-chemoradioselected advanced head and neck cancer patients (Aso et al., 2015; Hagiwara et al., 2016; Hagiwara et al. 2018; Table 6). Moreover, the association between CD44v9-containing isoforms overexpression/knockdown and chemoresistance *in vivo* has been studied (Table 2). Chemoresistance elevation in CD44v9-overexpressing gastric cancer xenografts was averted in the 5-FU and sulfasalazine (SAS)-treated groups, demonstrating tumor volume shrinkage when compared to both the control group and 5-FU group on days 21 and 28 (Miyoshi et al., 2018).

Overall, the implemented literature analysis pointed to a relationship between variant exon CD44v9 high expression levels and worse chemotherapy treatment outcomes of cancer patients. Cancer xenografts confirmed the negative relationship of variant exon v9 expression level with worse chemotherapy efficacy *in vivo*. The results demonstrate that CD44v9, in addition to CD44v6, may be important in chemotherapy resistance occurrence.

### 8.2 CD44 variant exon v9 biological functions in relation to cell death pathways

To date, CD44v9, alongside CD44v6, is a frequently studied variant exon compared to other clinically variant exons. It has been shown to influence apoptosis and, to a greater extent, ferroptosis in cancer cells (Table 3).

For instance, CD44v9-containing isoforms transfected to cancer cells demonstrated that CD44v9-overexpressing cells were significantly more resistant to apoptosis than CD44v3 and CD44s (Mielgo et al., 2006). However, blocking of CD44v9-containing isoforms with anti-CD44v9 antibodies (clone FW11.24) demonstrated their inability to restore the potential of cells to undergo apoptosis (Figure 6A; Mielgo et al., 2006). Therefore, we conclude that the noted effects may be explained by several reasons: 1) the antibodies used in the study do not properly bind to and interact with CD44v9, therefore apoptosis restoration was not detected and 2) this mechanism may be more supportive, rather than being a major one.

**FIGURE 8:**
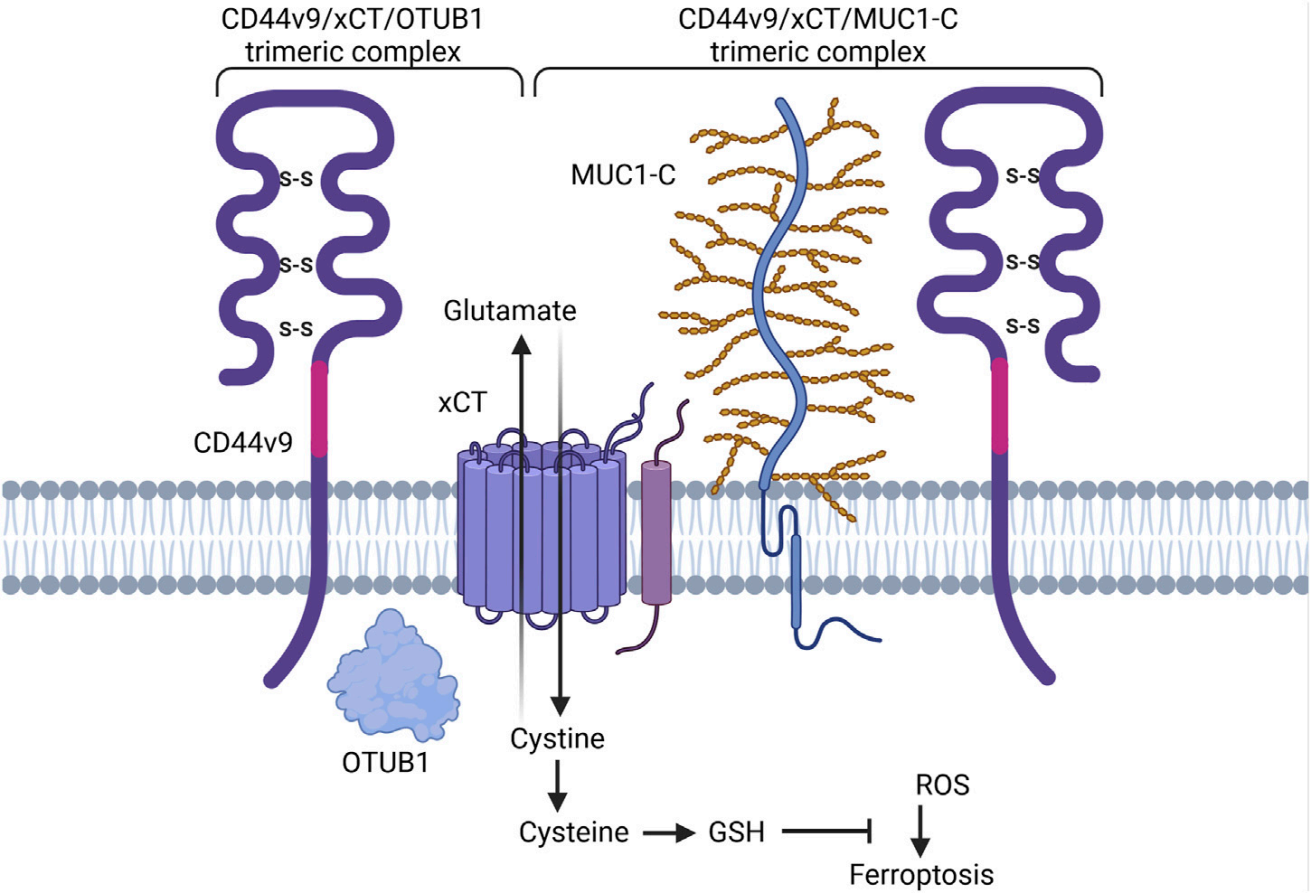
Schematic representation of CD44 variant exon v9 involvement in cell death pathways regulation. CD44v9 containing isoforms interact with and stabilize the xCT transporter, a key player in GSH synthesis and ferroptosis regulation. OTUB1 and MUC1-C provide additional stability of CD44-xCT complex (Created with BioRender.com).

Research conducted by Ishimoto et al. elucidated the interplay between CD44v9-containing isoforms and cystine-glutamate transporter xCT, a key player in glutathione (GSH) synthesis, reactive oxygen species (ROS) inactivation and ferroptosis regulation (Koppula et al., 2021). Their findings indicate that CD44 variant exons v8-v10 in gastrointestinal cancer (or CD44v9-containing isoforms in other types of cancers) interact with and stabilize the cystine transporter xCT, preventing it from being internalized and degraded as well as enhancing its capacity for downstream GSH synthesis and defense against ROS (Figure 8; Ishimoto et al., 2011). Interestingly, with the introduction of a S301A mutation containing consensus motifs for an N-linked glycosylation site in the variant region of CD44 (CD44v8-v10), CD44 variant isoforms failed to interact with xCT, thus underlining the importance of posttranslational modifications for CD44v9/membrane proteins interactions (Ishimoto et al., 2011). The precise molecular mechanisms underlying this interaction and stabilization are still under investigation but are thought to involve direct protein-protein interactions and possibly glycosylation-dependent processes.

Additional stability and degradation escape of CD44v9-xCT complex is carried out through interaction with ubiquitin thioesterase/otubain-1 (OTUB1), a deubiquitinating enzyme (DUB) that plays a vital role in regulating protein stability by removing ubiquitin molecules from target proteins, thereby preventing their degradation by the proteasome and as a result mediating ferroptosis (Liu et al., 2019; Figure 4A). OTUB1 has been shown to directly interact with and stabilize the xCT transporter: knockdown of OTUB1 led to growth suppression of mice tumor xenografts and reduced activation of ferroptosis (Liu et al., 2019). Interestingly, CD44 demonstrated the ability to stabilize xCT and OTUB1 interaction in an OTUB1-dependent manner. Moreover, the N-terminal domain of xCT is required for interacting with OTUB1, whereas the C-terminal domain of xCT is critical for binding CD44 (Liu et al., 2019). Although the direct interaction of CD44 and OTUB1 is much lower in comparison to OTUB1-xCT interaction, nevertheless, this three-protein complex is thought to stabilize xCT more effectively, possibly further enhancing chemoresistance.

Another player involved in CD44v9-xCT complex stabilization is mucin 1 (MUC1) – a transmembrane glycoprotein commonly overexpressed in a plethora of cancers including lung, colon, breast, pancreatic and ovarian cancers (Chen et al., 2021; Figure 4A). Hasegawa et al. uncovered the interaction between the oncogenic MUC1 C-terminal subunit (MUC1-C) and the CD44-xCT complex: it was demonstrated that MUC1-C extracellularly interacts with and stabilizes the xCT transporter and additionally interacts intracellularly with CD44 ICD (Hasegawa et al., 2016). It was demonstrated that treatment of MUC1-C^high^ cells with ferroptosis inducer/xCT inhibitor erastin was ineffective in ferroptosis execution; consequent receptor silencing led to the consecution of erastin-induced ferroptotic cell death (Hasegawa et al., 2016), uncovering an additional pathway for ferroptosis modulation.

Overall, CD44v9-containing isoforms contribute to ferroptosis resistance through mechanisms involved in cysteine metabolism, which is necessary for ferroptosis initiation. Additionally, apoptosis regulation has also been observed. Further research on these pathways will facilitate the development of CD44v9-targeted therapies to overcome chemoresistance and improve cancer treatment outcomes.

### 8.3 CD44v9 therapeutic strategies and clinical implications

Some approaches for CD44v9-exerted chemoresistance treatment entail the use of pharmacological inhibitors (Table 4). The sensitizing effect of SAS, a CD44v9-xCT signaling inhibitor, combined with cisplatin has been studied in pancreatic, hepatocellular and metastatic bladder cancer xenografts (Wada et al., 2018; Ogihara et al., 2019). It was demonstrated that combination treatment of xenografts with high expression of isoforms containing CD44v9 with SAS and cisplatin significantly decreased tumor growth and the number of tumor nodules compared to control cells, cisplatin alone and SAS alone. The current results indicate that such approaches may be useful for targeting CD44 variant isoforms to modulate chemoresistance and suggest alternatives/additives to standard chemotherapy regimens.

Notably, several cases have been reported surrounding the effect of CD44v9 expression on patient response to chemotherapy. For instance, in one study a patient treated with SAS for rheumatoid arthritis (RA), was further diagnosed with pT1 bladder cancer and underwent transurethral resection of bladder tumor (TURBT) (Takayama et al., 2016). They were further treated with MVAC and chemoradiotherapy due to the progression of the disease. With the appearance of a metastatic brain tumor, they underwent several courses of treatment with cisplatin and gemcitabine, and target lesions were classified as “complete response” shortly after the start of treatment. Interestingly, anti-CD44v9 staining of tumor tissue before TURBT and resected metastatic brain tumor unveiled a high expression and the absence of CD44v9 positive cells respectively, thus possibly attributing to the good clinical course outcome (Takayama et al., 2016).

In another study, a patient with prostate cancer underwent carboplatin-based chemotherapy (Takayama et al., 2016). During its administration, they were diagnosed with RA and PSA levels elevation was detected. Thus, alongside standard chemotherapy, SAS was administered, and surprisingly, PSA levels significantly decreased (by more than 50%) in just 2 weeks (Takayama et al., 2016). It should be noted that PSA levels further increased, although neuron-specific enolase (NSE) levels remained practically the same. Moreover, the prostate cancer tissue of the patient derived from needle biopsy and stained for CD44v9 demonstrated respective high expression levels, pointing to elevated resistance to chemotherapy (Takayama et al., 2016). Thus, CD44v9 expression levels may be of importance for favorable chemotherapy outcomes.

**FIGURE 9:**
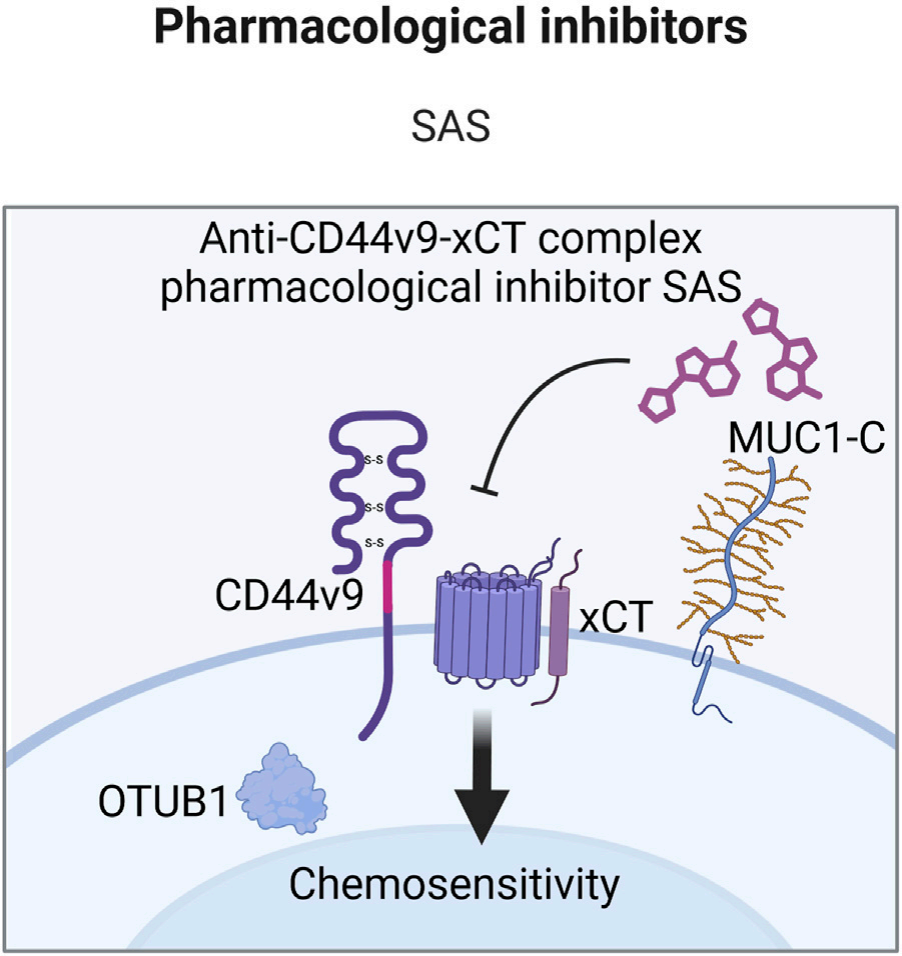
Clinical trials aimed at targeting CD44v9 containing isoforms (Created with bioRender.com).

Moreover, clinical trials have been conducted regarding the targeting of CD44v9-containing isoforms (EPOC1205 and EPOC1407) (Figure 9). A CD44v9 pharmacological inhibitor SAS demonstrated a substantial decrease in CD44 variant isoform expressing cells by more than 10% in half of the patients and a reduction of intratumoral GSH levels in 70% of patients after its administration in combination with CDDP in advanced gastric cancer. However, only three out of eleven patients achieved the stabilization of disease, questioning the efficacy of such treatment. Another study demonstrated the insufficient efficacy of SAS in combination with cisplatin on advanced gastric patients clinical parameters–obtained objective response was not detected (EPOC1407) (Uppaluri et al., 2017; Shitara et al., 2017; Table 5). Moreover, one out of four patients achieved the stabilization of disease for more than 4 months. Thus, the effects of CD44v9 targeting by SAS are under question.

## 9 Conclusion and perspectives

The interconnection of CD44 variant exons and total CD44 with cell death pathways execution and consequent chemotherapy resistance was explored in this review. Following the association of clinical parameters and prognostic potential of cancer patients with CD44 expression, it was identified that high levels of variant exons v3, v6, v7, v9 and v10 are significantly associated with shorter progression-free, disease-free and overall survival, whereas CD44 variant exons v4, v5 and CD44s had no association (Table 6). The studies with overexpression and knockdown of CD44 variant isoforms confirmed these associations for CD44 variant exons v6 and v9, as well as total CD44 with the help of *in vivo* experiments (Table 2).

To date, variant exon CD44v6 containing isoforms effect on chemoresistance has been studied the most out of all variant exon containing isoforms of CD44. CD44v6 can modulate apoptosis and autophagy resistance through growth factor presentation and direct interaction with coreceptors on the cytoplasmic membrane, thus being a promising cell death-potentiating therapeutic target (Table 3). As seen in Table 5, clinical studies predominantly aim to target cells with high expression levels of CD44v6-containing isoforms. Current clinical studies include both ineffective and ongoing therapies. Ineffective therapy was observed for antibodies targeting CD44v6, but a pharmacological inhibitor AMC303 and anti-CD44v6 CAR-T cells have demonstrated exemplary safety. Further analysis concerning clinical effects is expected.

Another commonly studied variant exon is CD44v9. CD44v9 is predominantly involved in ferroptosis regulation, a recently discovered type of cell death (Table 3). Modulating the CD44v9-ferroptosis axis is relevant for patients resistant to classical cell death pathways such as apoptosis. In addition, cancer cells were shown to have increasing levels of intracellular iron, thereby making ferroptosis induction a potential targeting approach. Interestingly, CD44 was shown to be involved in HA-bound iron uptake and consequent expression self-potentiation. This may be important for drug resistance alleviation since increased iron uptake sensitizes cells to ferroptosis. Some clinical studies have shown positive outcomes with the addition of SAS to standard therapy (mainly to cisplatin), whereas others point to an absence of results (Table 5). Clinical studies surrounding CD44v9 targeting include trials with not so prominent effects on patient survival outcomes, although at the tumor cellular level, there is a decrease in CD44v9^+^ cells. Nevertheless, the mentioned studies suggest that the development of alternative CD44v9-targeting approaches may be relevent. Currently, existing trials aim to target CD44v9 only with a pharmacological inhibitor of CD44v9-xCT complex SAS.

CD44v3, the least studied variant exon of CD44 concerning clinical outcomes of patients, is involved in apoptotic cell death pathway regulation through a surprisingly large number of molecular mechanisms (Table 3). Such mechanisms include growth factor binding and presentation to receptors on the plasma membrane with the help of HS moieties and the engagement of epigenetic signaling. No therapeutic substances targeting CD44v3 have been tested in clinical settings, and we propose that this may be due to the complex nature of the CD44v3 structure. Notably, a recent study has established a novel monoclonal antibody, C44Mab-6, that recognizes a peptide sequence excluding an HS-modified sequence in the CD44 variant-3-encoded region. Testing this antibody *in vivo* and in clinical settings may be a promising approach.

Studies have also been conducted regarding the total CD44 effect on chemotherapy resistance. Total CD44 is involved in several cell death-modulating signaling pathways either by direct interaction with receptor proteins on the plasma membrane or through the changes in its signaling pathways upon interaction with its ligands (Table 3). A distinctive feature of total CD44 is its inclusion of standard and variant isoforms. Studies on chemotherapy treatment outcomes of patients point to a predominant involvement of CD44 variant isoforms, but not CD44s, in chemoresistance. Additionally, clinical trials with total CD44 targeted therapies have shown success in ovarian and oral cavity squamous cell carcinomas and the lack of success in a study that included metastatic and/or locally advanced CD44-expressing malignant solid tumors (Table 5). This may indicate that total CD44 targeting may be successful for several individual cancers but generally seems ineffective over several cancers. Thus, the biological effect of total CD44 may be exerted due to CD44 variant exon-containing isoforms, and we believe that their targeting may be more beneficial for targeting chemoresistant cancer cells.

Importantly, CD44 has been identified as the most common CSC marker, and is tightly associated with stemness properties of CSCs. This topic has been extensively reviewed, including in several recent publications (Jaggupilli and Elkord, 2012; Skandalis et al., 2019; Wang et al., 2018; Yan et al., 2015). Generally, CSCs are known to have the capacity to self-renew and differentiate, and to exhibit resistance to drug and radiation (Wang et al., 2018). Surprisingly, not only CD44 variant isoforms, but also CD44s have been identified as CSC markers in prostate, colon, gastric, pancreatic, ovarian and breast cancers (Bhattacharya et al., 2018; Kimura et al., 2013; Lau et al., 2014; Li et al., 2014; Todaro et al., 2014; Zeng et al., 2013; Zhang et al., 2019). This data once again underscores the functional complexity of CD44 splice variants not only in tumor cells, but also in CSCs. Nevertheless, the mechanisms of cell death resistance mentioned in our review, for total CD44 as well as CD44v3, CD44v6 and CD44v9 were demonstrated to be the same in CSCs. However, other mechanisms may exist that have not yet been described in the current literature. Additionally, the CD44 isoforms that serve as CSC markers in a particular tumor may not necessarily be the predominant isoforms expressed throughout the entire tumor. At the same time, therapeutic strategies aimed at overcoming resistance usually target the predominantly expressed isoforms, which additionally have prognostic value. While such strategies can effectively overcome the resistance of the majority of tumor cells, they may fail to address CSCs, potentially leading to tumor recurrence or metastasis. Therefore, therapies aimed at suppressing CD44 in CSCs, or combination therapies targeting both the predominant tumor isoforms and CSC-specific marker may prove more effective in overcoming resistance and preventing recurrence or metastasis.

With that being said, despite the fact that there are studies aiming to target CD44, the results of clinical studies to date are ambiguous. This can be due to several reasons, one being the high variability of CD44 across different cancers. Indeed, besides variant exons, CD44 with only constant exons is expressed at reasonably high levels in tumor tissues. Since several developing therapeutic substances target total CD44, including both CD44s and variant isoforms, this can possibly lead to nonsignificant targeted therapy outcomes. Further research is necessary for precise and effective therapeutic substance development. We propose that targeting proteins involved in exon alternative splicing may possibly be the solution. The mechanisms by which variant isoforms of CD44 are formed by alternative splicing are described in detail in the recent review (Maltseva and Tonevitsky, 2023). For instance, splicing factor SRSF1, promoting the switch from variant isoforms to CD44s, was shown to inhibit autophagy (Bangming et al., 2024). Therefore, modulation of splicing factors’ activity may allow the evasion of high variability of CD44 proteins and enhance cell death pathways. However, it is undoubtedly important to remember that splicing factors have many targets, and it is important to test the overall effect of their activity modulation on cells. For this reason, an approach aimed at inhibiting not the activity of the splicing factors but the efficiency of their binding to CD44 mRNA, e.g., using splicing-switch oligonucleotides (Maltseva and Tonevitsky, 2023), may be even more promising and have fewer side effects.

In conclusion, CD44 isoforms containing variant exons play an intricate role in cancer chemoresistance by selectively modulating key cellular death pathways, including apoptosis, autophagy and ferroptosis. The results of completed clinical studies targeting CD44 isoforms containing variable exons are ambiguous. Therefore, the need to understand the role of CD44 in the mechanisms of chemoresistance remains. A deeper understanding could allow the creation of a more targeted therapeutic drug that specifically targets tumor cells and improves patient outcomes in cancer treatment.

## Supplementary material

SUPPLEMENTARY FIGURE S1 Expression profile of CD44 splice variants (both experimentally confirmed and predicted isoforms) in cancer patients. Created by www.gepia2.com.

## Glossary

5-FU: 5-fluorouracil
ATG: Autophagy-related protein
**CD44** ^**kd**^: CD44 knockdown
CD44v2: CD44 variant exon v2
CD44v3: CD44 variant exon v3
CD44v5: CD44 variant exon v5
CD44v6: CD44 variant exon v6
CD44v7: CD44 variant exon v7
CD44v9: CD44 variant exon v9
c-Met: Mesenchymal epithelial transition factor receptor
CQ: Chloroquine
DSS: Disease-specific survival
DUB: Deubiquitinating enzyme
ECD: Extracellular domain
EGF: Endothelial growth factor
ERBB2: Receptor tyrosine-protein kinases 2
FOLFOX: Folinic acid, 5-FU and oxaliplatin
GSH: Reduced glutathione
HA: Hyaluronan
HABD: HA-binding domain
HGF: Hepatocyte growth factor
HMW: High molecular weight
HS: Heparan sulfate
ICD: Intracellular domain
IHC: Immunohistochemistry
ITG: Integrin
LC3: Microtubule-associated protein 1A/1B-light chain 3
Mer/NF2: Merlin/neurofibromin-2
MMPs: Metalloproteinases
MST1/2: Mammalian STE20-like kinase 1 and 2
mTOR: Mammalian target of rapamycin
MUC1: Mucin 1
MUC1-C: Mucin 1 C-terminal subunit
NCCD: Nomenclature Committee on Cell Death
NLS: Nuclear localization signal
NSCLC: Non-small cell lung cancer
NSE: Neuron-specific enolase
OPN: Osteopontin
OS: Overall survival
OTUB1: Ubiquitin thioesterase/otubain-1
PAAD: pancreatic cancer
PAR1b: Polarity-regulating serine/threonine kinase
PCD: Programmed cell death
PHF8: PHD finger protein 8
PRAD: prostate cancer
PSA: Prostate antigen
QC: Quinacrine
RA: Rheumatoid arthritis
RFS: Recurrence-free survival
RNA-seq: RNA sequencing
ROS: Reactive oxygen species
RTKs: Receptor tyrosine kinases
SAS: Sulfasalazine
SMCs: Smooth muscle cells
TCGA: The Cancer Genome Atlas
TG2: Transglutaminase 2
TMD: Transmembrane domain
TURBT: Transurethral resection of bladder tumor
VEGFR-2: Vascular endothelial growth factor receptor 2
YAP: Yes-associated protein
