# Supplementary figures and images for: CD44 variant exons induce chemoresistance by modulating cell death pathways
Source: Front Cell Dev Biol. 2025 Mar 6;13:1508577. doi: 10.3389/fcell.2025.1508577 (PMC11924683; doi:10.3389/fcell.2025.1508577)

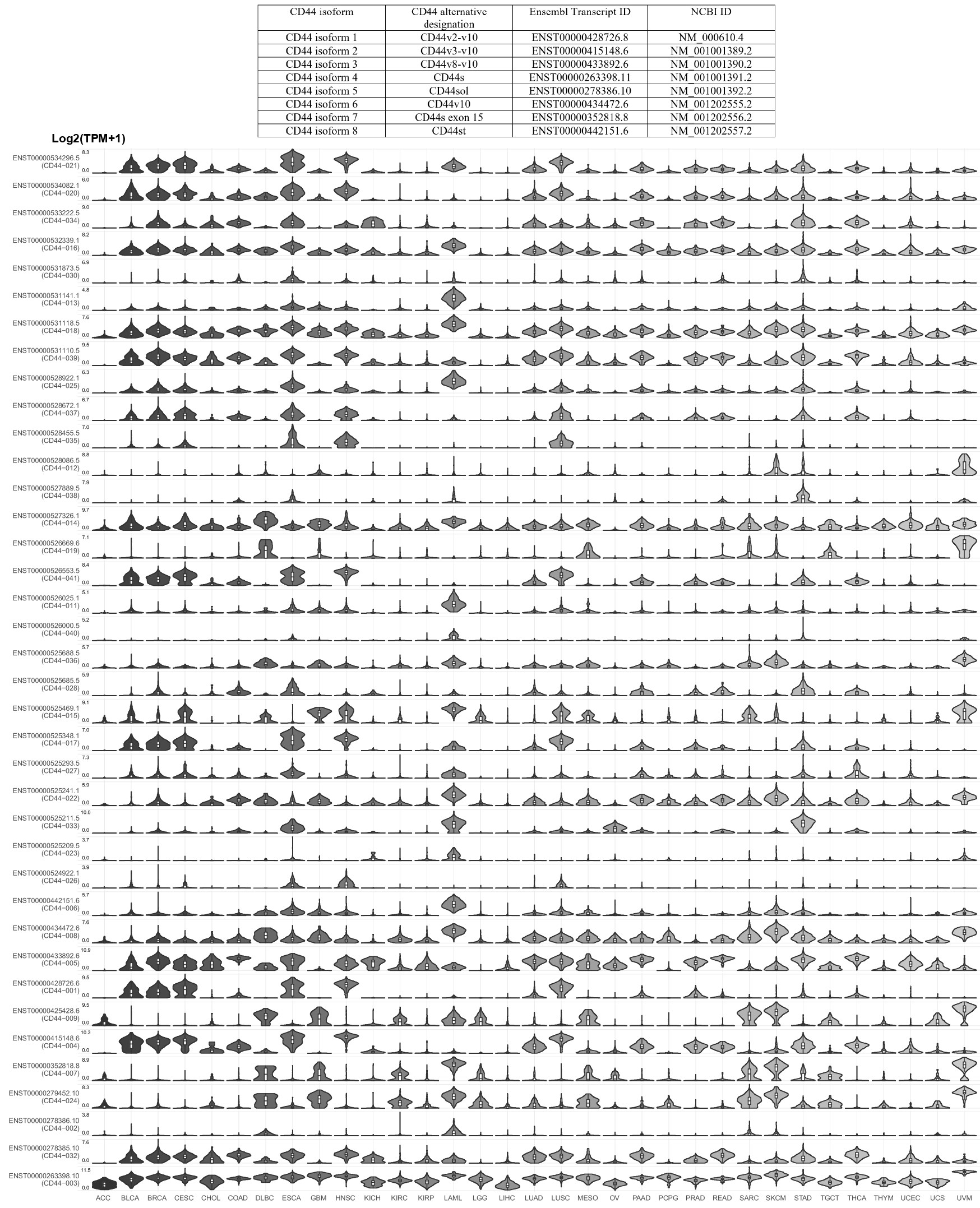

Supplement: Supplementary file 1 [file Image1.jpeg]
